# Oxidative Stress and Maxi Calcium-Activated Potassium (BK) Channels

**DOI:** 10.3390/biom5031870

**Published:** 2015-08-17

**Authors:** Anton Hermann, Guzel F. Sitdikova, Thomas M. Weiger

**Affiliations:** 1 Department of Cell Biology, Division of Cellular and Molecular Neurobiology, University of Salzburg, Salzburg 5020, Austria; E-Mail: thomas.weiger@sbg.ac.at; 2 Department of Physiology of Man and Animals, Kazan Federal University, Kazan 420008, Russia; E-Mail: sitdikovaguzel@gmail.com

**Keywords:** Maxi calcium-activated potassium ion channels, BK channels, KCNMA1, redox mechanisms, oxidation, hypoxia, ischemia

## Abstract

All cells contain ion channels in their outer (plasma) and inner (organelle) membranes. Ion channels, similar to other proteins, are targets of oxidative impact, which modulates ion fluxes across membranes. Subsequently, these ion currents affect electrical excitability, such as action potential discharge (in neurons, muscle, and receptor cells), alteration of the membrane resting potential, synaptic transmission, hormone secretion, muscle contraction or coordination of the cell cycle. In this chapter we summarize effects of oxidative stress and redox mechanisms on some ion channels, in particular on maxi calcium-activated potassium (BK) channels which play an outstanding role in a plethora of physiological and pathophysiological functions in almost all cells and tissues. We first elaborate on some general features of ion channel structure and function and then summarize effects of oxidative alterations of ion channels and their functional consequences.

## 1. Introduction

Ion channels play a pivotal role for the functioning of any cell in the animal as well as in the plant kingdom. An important class of ion channels is the family of potassium (K^+^) channels, they are not only in charge of the membrane resting potential or the repolarization of the action potentials, but also control cell proliferation or transmitter/hormone release, to name a few. A subgroup of K^+^ channels are the so called calcium (Ca^2+^) activated K^+^ channels which need either an increase of Ca^2+^ at their intracellular face to open or a combination of Ca^2+^ and voltage to function properly. Maxi Ca^2+^ activated K^+^ channels, also named BK channels which constitute a subgroup of Ca^2+^ activated K^+^ channels, are in the focus of our review. These channels modulate a number of physiological events, like blood pressure, smooth muscle relaxation or electrical tuning of hair cells in the cochlea and have a leading role in many pathophysiological conditions such as epilepsy or the behavioral response to alcohol, to give only a few examples. Oxidative stress on the other side is a physiological byproduct of any aerobic metabolic process and as such common for cells to deal with. Oxidation modulates many pathways in the cell including activity of ion channels like BK channels. The modulatory actions of oxidative stress on BK channels will be in the focal point of this paper. First, in Section 2, we will address general properties of ion channels and in particular the qualities of BK channels. In Section 3, we will specify what we mean by the term oxidative stress, while Section 4 will deal with the impact of redox modulation of BK channels. At the end, we will discuss these findings in the light of their clinical relevance and conclude with perspectives and vistas.

## 2. Ca^2+^-Activated K^+^ Channels (K_Ca_)

### 2.1. General Introduction to Ion Channels

The task of cell membranes (plasma/organelles) is to separate cellular compartments, such as extra- and intracellular or extra- and intra-organelle areas to allow for proper performance of biochemical processes. Since the lipid bilayer membranes are almost impermeable for ions, tunnel proteins are inserted to allow for communication between compartments for electrical and chemical signaling. In addition membranes themselves are sites for processing biochemical reactions. Readers familiar with ion channels may omit this section on general features of ion channels.

Since cellular ion channels form minute pores across membranes they are suitable for the passage of ions, in particular monovalent sodium (Na^+^), K^+^, protons (H^+^), the divalent cation Ca^2+^ or the anion chloride (Cl^−^). These ions are differently concentrated across cell membranes and various mechanisms are involved in the separation of ions, *i.e*., (a) active transport systems, which directly consume energy (adenosine triphosphate, ATP) to transport ions against a concentration gradient; (b) transport systems which do not directly consume ATP but use the concentration gradient across the membrane as driving force; or (c) the redistribution of ions due to fixed negative charges provided mostly by intracellular proteins (Donnan equilibrium). In essence, this creates an electro-chemical gradient for each ion which provides for a driving force since for example at the membrane resting potential, endowed in all living cells, none of the ions is at equilibrium [1].

Most ion channels are gated, which means they contain an intrinsic mechanism which allows for either a closed or open configuration. Once the pore is open ions can flow according to their electro-chemical gradient and as moving charges produce a current which provides a potential difference across the membrane. Hence, in difference to our technical current which is brought about by a flow of electrons, living cells use ions as charge carriers.

An enormous amount of knowledge about the structure and function of ion channels has been gathered over the last decades. This development was based on the seminal work by Hodgkin and Huxley (1952) [2]. They provided the basis for proper measurement of membrane potentials and they measured the ionic current flow across cell membranes by application of the “voltage clamp” technique which inspired generations of scientists. Only about 30 years later Neher and Sackmann (1981) [3], by installing the “patch clamp” method, succeeded to measure the flow of ions through single channels in native cell membranes. This technique, in fact allows us to visualize a protein at work. Again, 20 years later, MacKinnon and his group (1998) [4] succeeded to combine molecular-, electrophysiological techniques and structural analysis to develop a 3-dimensional scheme of an ion channel and its functions. It is clear now that there are many more types of ion channels than previously imagined. Although not all details concerning ion channels have been resolved to date, however, the knowledge gathered by scientists on structure and function over the last decades is overwhelming.

In general, ion channel proteins consist of hydrophobic amino acid α-helices inserted into the membrane lipid bilayer connected by hydrophilic amino acid linkers. Cation channels usually contain four domains with two, four, or seven α-helix segments. In some channels amino acids built one continuous fibrous structure (*i.e*., Na^+^-, Ca^2+^ channels), while others consist of four separate domains which form a tetrameric channel within the membrane (*i.e*., K^+^ channels). In most channels “α-subunits” form the pore for the passage of ions. Attached to the α-subunits are in most cases a variety of other proteins—called auxiliary subunits (β, γ, ε, δ) [5], or special channel specific proteins, and/or enzymes, such as kinases, phosphatases or heme [5,6]. To classify ion channels by their mechanism of activation (gating) has been considered as a proper means.

**Figure 1:**
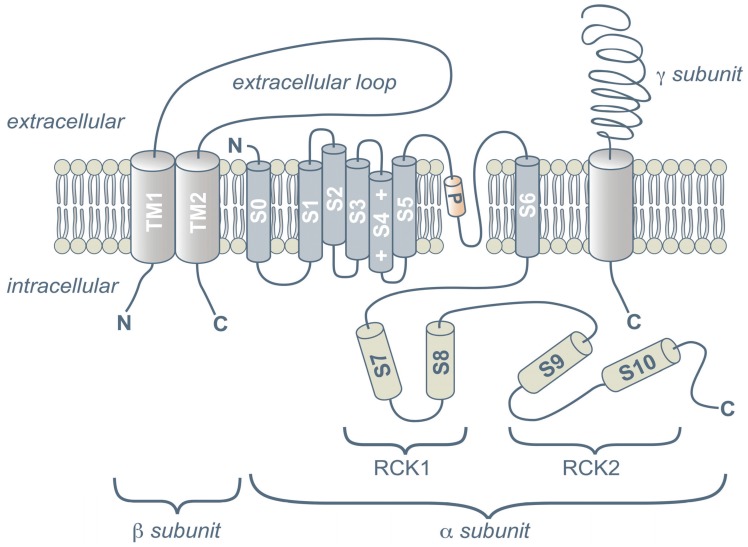
Schematic structure and membrane topology of maxi calcium-activated potassium (BK) channel α, β and γ subunits (adapted from [44]). See text for a discussion of the structure.

The structure and function of ion channels has been summarized in several reviews [7,8,9,10,11]. The today’s extensive realm of ion channel structure and function is brought about by the separation into many special topics, such as for Na^+^, Ca^2+^, K^+^, Cl^−^ [12,13,14,15,16] channels. The field of ion channels developed immensely and we therefore limit our brief introduction to maxi calcium activated K^+^ or BK channels which are the main focus of this review. K^+^ channels can be devoid of auxiliary subunits but usually have various types of β-subunits, *i.e*., BK channels consist of four types of β subunits (β1–β4), and a set of γ-subunits (Figure 1). These subunits are of particular interest because of their potential to diversify the transport properties and distribution of channels in various cells and tissues. Attached to the α-subunits these auxiliary subunits cause a variety of different effects on channel gating, on current kinetics or conductance, on trafficking the α-subunit to the cell membrane, link to intracellular cytoskeleton and extracellular matrix proteins, modulation of channel expression or they provide for binding of drugs.

In general, the activity of ion channels which composes the ion current and hence the potential across the membrane is governed by three factors: the number of active channels (N), the channels in the open state (Po = open probability) and the single channel current (i). This can be summarized by the relationship: I_tot_ = N Po i, where I_tot_ is the total current, N is the number of functional channels and i is the single channel current. In most cases Po is modulated by ligands or drugs.

### 2.2. Types of K_Ca_ Channels

K_Ca_ membrane permeability was first described from experiments using erythrocytes where the presence of internal Ca^2+^ increased the permeability of the cell plasma membrane to K^+^ [17]. It took more than a decade to transfer this idea to other cells and to further investigate this phenomenon in more detail. To date a variety of K_Ca_ channels have been described in a great variety of excitable and non-excitable cells of many species and are mainly defined by their biophysical and pharmacological properties.

The K_Ca_ channel family contains eight members according to the sequence homology of transmembrane hydrophobic segments [18] with four subfamilies: **BK** (or K_Ca_1.1, Slo1, Maxi-K, *KCNMA1*), **SK** (K_Ca_2.1 (SK1, *KCNN1*), K_Ca_2.2 (SK2, *KCNN2*), K_Ca_2.3 (SK3, *KCNN3*), **IK** (K_Ca_3.1 (IK_Ca_1, SK4, *KCNN4*) and **other subfamilies** (K_Ca_4.1 (*KCNT1*), K_Ca_4.2 (SLICK, Slo2.1, *KCNT2*), K_Ca_5.1 (Slo3, *KCNU1*). These channels generally consist of four α-subunits with six membrane-spanning α-helices (segments) per domain (S1–S6) containing the K^+^ pore. Both, N- and C-terminal are usually at the internal, cytosolic side of cells. In addition, Slo 1 (BK) and Slo 3 channels have one more transmembrane helix (S0) which locates the N-terminus to the external side (see Figure 1 and section BK channels). Not all of these channels are responsive to Ca^2+^—since some are activated by Na^+^ (Slo 2) or Cl^−^ (Slo 3).

### 2.3. Maxi Calcium-Activated K^+^ Channels (BK)

BK channels are expressed by a single gene. The conductance of single BK channels is up to 300 pS and hence comprises the largest single-channel conductance of all K^+^ channels. To date a vast amount of information has been gathered regarding their biophysical, structural and functional, physiological, pathophysiological and pharmacological properties (recently reviewed in [19,20,21,22,23,24,25,26,27,28,29,30,31,32,33,34,35,36,37]). BK channels are usually synergistically activated by both—Ca^2+^/Mg^2+^—metal ions and by membrane voltage. The channels can be also activated in the absence of Ca^2+^ but then require an extremely large depolarization of 100–200 mV which is not physiologically relevant but important for the understanding of some biophysical properties of these channels. BK channel α-subunits consist of a total of seven transmembrane segments with a S0 segment preceding the six transmembrane segments (S1–S6). This renders the N-terminus (amino terminal) at the extracellular side of the membrane (Figure 1). The transmembrane segments (S1–S4) consist of various charged amino acid residues which confer voltage sensitivity to the channels. The S0 segment appears mainly required for interaction of α-subunits with auxiliary β-subunits as well as for targeting the channels to the plasma membrane. As in other voltage dependent K^+^ channels, four pore-forming loops between the segments S5-S6 of each α-subunit configure the ion selectivity filter. The large C-terminus (carboxyl terminal) comprises about two thirds of the α-subunit protein and contains various binding sites for kinases, phosphatases and negatively charged Ca^2+^ binding sites as well as two so called RCK-domains (regulatory domain of K^+^ conductance) (Figure 1).

Multiple splice variants of the α-subunit have been identified resulting in a great variety of channel properties in various cell types [38]. Through alternative splicing the pore forming α-subunit contains at its C-terminus a cysteine-rich 59-amino-acid insert between RCK domains called stress-axis regulated exon (STREX). STREX exon expression is under physiological conditions initiated by the stress-axis adrenocorticotropic hormone [39]. STREX inserts cause BK channels to activate at more negative potentials, enhance activation and decrease deactivation which leads to increased repetitive firing of action potentials. STREX inserts can also be artificially produced by growing cells in phenol red which causes a significant increase in channel sensitivity to inhibition by oxidation [40]. Phenol red, which is routinely used as a pH indicator in tissue culture media, bears structural resemblance to some nonsteroidal estrogens and has significant estrogenic activity [41]. Further findings indicate that maxi-K channel transcripts are differentially spliced by 17β-estradiol, which may contribute to changes in isoform expression [42] and may also lead to STREX expression in GH3 cells which carry estrogen receptors [43].

Specific blockers of the channels are tetraethylammonium (TEA) at submillimolar concentrations [45,46], iberiotoxin (from scorpion) or paxilline (mycotoxin from *Penicillium paxilli*) [47,48]). BK channels can be expressed together with other types of K_Ca_ channels (SK or IK) [49] or in particular with Ca^2+^ channels [50,51] which makes them important modulators of electrical discharge activity and synaptic transmission. Ca^2+^ as a major player in cellular signaling is involved in a large variety of cell functions and links cell excitation to cell metabolism and gene expression.

BK channels are associated and modulated by a wide variety of intra- and extracellular factors, such as auxiliary subunits (β, γ), Slobs (slo binding protein), phosphorylation, gasotransmitter action (nitrosylation, carboxylation, sulfhydration) or by redox mechanisms [11,44,52,53,54,55,56,57,58,59,60,61,62,63,64,65,66,67]. The BK α-subunits assemble 1:1 with four different auxiliary types of **β-subunits** (β1, β2, β3 or β4) (Figure 1). β-subunits are members of the protein superfamily of oxidoreductases [68,69] which contain 191–235 amino acids [70,71,72,73]. BK β-subunits consist of two transmembrane segments (TM1, TM2) with both, the N-terminal and the C-terminal located intracellularly. In different tissues four different genes can be expressed, as in smooth muscle, adrenal chromaffin cells or in neurons. β-subunits are involved in modifying voltage sensitivity, current kinetics and/or pharmacological properties of BK channels [65,70,74]. β-subunits are also responsible for tissue specificity, they can alter channel activity by activation of protein kinases, confer hormone (estradiol) activation, and alter toxin binding to the channels [75]. They are involved in current inactivation by a flexible N-terminal chain and ball structure which blocks the channel’s pore [76,77]. In the brain, beta 4-subunits for example, inhibit BK channel activation and slow down channel kinetics [65,78,79,80], confer resistance to peptide blockers such as charybdotoxin and iberiotoxin [65,81,82] or protect against temporal lobe seizures [79].

Various leucine-rich repeat containing proteins (LRRCxx), have been identified and designated as auxiliary **γ-subunits** (Figure 1) [66,83,84]. These proteins, which are clearly distinct from β-subunits, interact with BK channel α-subunits. Various types of γ-subunits cause in the absence of Ca^2+^ a negative shift of up to ~140 mV in the voltage dependence of the BK channel activation, *i.e*., the channels open at voltages near the membrane resting potential. This way they exert interesting physiological functions in a great variety of tissues, such as the nervous system, skeletal muscle, and adrenal glands. For example, in fetal nervous tissue using knock-down studies, γ1-subunits appear to participate in governing neuronal excitability in the early development of the brain [83] and in rat cerebral arterial smooth muscle LRRC26 constitutes functional γ-subunits involved in vaso-regulation [84]. In non-excitable cells, such as in salivary glands, prostate, testes or the airway epithelia, the hyperpolarizing actions of γ-subunits provide for an increase of the driving force for Ca^2+^ via non-voltage dependent (ligand gated) Ca^2+^ channels. It was suggested that γ-subunits may offer therapeutic potential targets as BK channel openers for the development of new BK channel–activating drugs in the treatment of various diseases [66].

A further class of proteins, called Slo binding proteins (**Slobs**) can attach to and modulate BK channels [85,86,87,88]. Slob 57, for example, shifts the voltage dependence to more depolarized voltages and causes faster closure of the channels [87]. Furthermore, Slob exerts a diurnal cycle *in vivo* [89] indicating that BK channel activity changes as a function of day time imparting circadian rhythmicity to neurons. Other Slobs like Slob71 or Slob 53, shift the voltage dependence to less depolarized voltages but have no effect on channel kinetics [88].

Some BK channels can be activated by stretch or pressure. These stretch-activated BK channels (SAKCaC) [90,91] are expressed in a variety of tissues such as in myocytes or neurons and modulate vascular smooth muscle tone and endocrine cell secretion. A STREX insert between RCK1 and RCK2 domains at the channel’s C-terminal α-subunit is indicated to confer stretch sensitivity to the channels [90,92]. However, other BK channels lacking the STREX insert still remain sensitive to membrane stretch suggesting that additional structures of the channel may be responsible for mechanical coupling to the cell membrane [93].

### 2.4. Function of BK Channels in Norm and Pathology

BK channels contribute to various functions, such as controlling electrical discharge activity of nerve and muscle cells. Since the opening of K^+^ channels drives the membrane potential towards the K^+^ equilibrium potential, this will result in hyperpolarization of the membrane resting potential which will lead a nerve cell away from excitation. On the other hand, it may speed up the repolarization of action potentials, which makes their duration shorter and more action potentials per time can be generated. At the synapse the duration of the excitation, *i.e*., duration of the action potential is translated into the amount of opening of Ca^2+^ channels, influx of Ca^2+^ into the cell and an increase of the internal Ca^2+^ concentration which directly relates to the quantity of transmitter release or hormone secretion [94]. Similar mechanisms play a role in vaso-regulation, auditory tuning of the hair cells, in erectile processes and participate in mediating drug actions such as ethanol or acetaldehyde. In circadian rhythm generation BK channels are expressed in neurons of the supra chiasmatic nucleus during night and are removed during the day causing silencing or excitation, respectively [95]. Targeting of BK channels to appropriate membranes is important for the proper functioning of cells and organelles. Trafficking to and expression of BK channels in the plasma membrane has been found to be regulated by distinct splicing motifs located within the intracellular C-terminal RCK domains [96]. In particular a splice variant that excluded these motifs prevented cell surface expression of BK channels and suggests that such a mechanism impacts physiology and pathophysiology.

BK channels are not only present in the plasma membrane of cells but are also located in the membranes of cellular organelles such as mitochondria, nucleus, endoplasmic reticulum or the Golgi apparatus (reviewed in [97]). Information on the function and regulation of BK channel in organelles is at its infancy. A recent report indicates that nuclear BK channels (nBK) regulate gene expression of hippocampal neurons in a synaptic activity-evoked, Ca^2+^ dependent manner, suggesting that nBK channels play an important role as modulator and molecular linker of neuronal activity dependent functions and nuclear Ca^2+^ [98]. Since most information is available from mitochondria we will briefly discuss these channels in a later section.

Channelopathies are caused by mutations at one or more amino acids of the channel protein which may lead to dysfunction of cells and organs. These mutations can occur at any site of the channel protein, the pore, the voltage sensor, or the inactivation structures, including auxiliary subunits. Channelopathies affect all kinds of channels (voltage-, ligand-, mechano-gated, *etc.*), and hence are involved in a multitude of disorders, such as epilepsy, stroke, paroxysmal movements, cerebellar ataxia, hearing loss, autism, asthma mental deficiency, myotonia, heart diseases, hypertension, cystic fibrosis, bladder or gastric hypermotility, erectile dysfunctions, *etc.* (for recent reviews see [25,32,99,100,101,102]). BK channel mRNA expression is lower in the prefrontal cortex of schizophrenic, autistic, and mentally retarded persons [103,104]. Mutation at the α-subunit which is associated with idiopathic generalized epilepsy and paroxysmal dyskinesia [105] appear to result from augmented Ca^2+^ sensitivity at the RCK1 binding site together with mutations at the brain specific β4-subunit. Mutant BK channels being more sensitive to Ca^2+^ were found to increase excitability in humans by causing a more rapid repolarization of action potentials which in turn limits the amount of Ca^2+^ flowing into the cell. An enhanced repolarization favors a faster removal of inactivation from Na^+^ channels and thus lets neurons fire at a higher frequency. A nonfunctional β4-subunit which under wild type conditions broadens the actions potential on the other hand also leads to faster action potential repolarization. Eventually this results in an increased discharge of action potentials which may lead to epilepsy, paroxysmal movement disorders, or alcohol dependent initiation of dyskinesias [79,106]. Although depletion of BK channels in the brain appears not to be lethal it produces a variety of deficiencies as indicated above.

This introduction to the realm of ion channels as well as BK channels in detail was intended to provide some background for (a) getting to know and summarizing the structure and function of these proteins and for (b) providing a basis for the following elaboration of their modulation by oxidative stress.

## 3. Oxidative Stress

**Figure 2:**
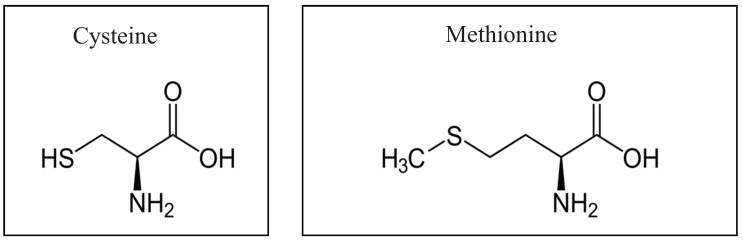
Structure of the sulfur containing amino acids cysteine and methionine.

Oxidative stress in general has been introduced by other authors of this special issue, defined, interpreted and generally covered and therefore will not be further discussed. In this section we intend to summarize some aspects of redox effects and oxidative stress on BK channels. Reactive oxygen species (ROS) are involved in a magnitude of oxidative stress modulations but also in the physiological regulation of a great variety of proteins and cell functions as outlined in various reviews [34,61,97,107,108,109,110,111,112,113,114,115,116,117]. The sulfur containing **cysteine** (Cys or C) and/or **methionine** (Met or M) residues (Figure 2) within proteins are preferred targets of redox modulation, *i.e*., reversible oxidation/reduction. Cysteines can be oxidized at their thiol groups where two cysteine molecules link to form disulfide bonds internally within the protein or with other proteins. For experiments, cysteine specific reagents 5'5-dithio-bis(2-nitrobenzoic acid) (DTNB), methanethiosulfonate ethylammonium (MTSEA) or p-chloromercuribenzoic acid (PCMB) have been used. Biochemically, disulfide bonds are readily reduced, *i.e*., by dithiothretiol (DTT) or mercaptoethanol (β-ME) to convert them back to thiol cysteines. *In vivo* reversibility of cysteines is brought about by antioxidants such as the tripeptide glutathione (GSH) or the small protein thioredoxine (TRX) which is present in all organisms [118]. Methionine can be preferentially oxidized by agents such as chloramine-T (Ch-T) under physiological conditions or by *N*-chlorosuccinimid (NCS) [119]. Methionine residues have been hypothesized to function as endogenous antioxidants in proteins [119]. Oxidation leads to polar methionine sulfoxide (Met-O) which can be reversed by methionine sulfoxide reductases (MSRA). Some oxidizing/reducing (ROS) agents are listed in Table 1.

**Table 1:** Reactive oxygen species (ROS) agents, reducing agents and gasotransmitters that are produced by cells and/or are experimentally used chemicals for modulating BK channels.

Oxidizing/Reducing agents at BK channels			
Chemical nomenclature	Abbrev./ Formula	Company	Notes/literature
**Oxidizing agents**			
Hydrogen peroxide	**H_2_O_2_**	Fisher Scientific; Sigma	commonly used oxidizing agent. H_2_O_2_ is naturally produced by the cell metabolism, is membrane permeable and relatively stable [120,121].
Superoxide anion	**O_2_^−•^**	Sigma	radical; range of 10^−11^ to 10^−10^ M; [122] antagonist: superoxide dismutase.
Glutathione disulfide	**GSSG**	Sigma	physiological oxidized form of GSH [123].
Chloramine-T	**Ch-T**	Sigma	oxidizes methionine [119]; oxidation leads to polar methionine sulfoxide (Met-O), reversed by methionine sulfoxide reductases [109].
*N*-chlorosuccinimide	**NCS**	Sigma	oxidizes methionine [119].
4,4'-dithiodipyridine	**4,4DTDP**	Sigma	[124]
2,2'-dithiodipyridine	**2,2DTDP**	Sigma	[125,126]
5'5-dithio-bis(2-nitrobenzoic acid	**DTNB**	Sigma	cysteine-specific reagent [109,123,127].
(2-Aminoethyl) methan-ethiosulfonate hydrochloide	**MTSEA^+^**	Toronto Research	cysteine-specific, covalently modifies free thiol groups [125].
2-(trimethylammonium) ethyl methanethiosulfonate, bromide	**MTSET**	Toronto Research	[128]
p-chloromercuribenzoic acid	**pCMB**	Sigma	cysteine-specific [109].
Sodium (2-sulfanatoethyl) methanethiosulfonate	**MTSES**	Toronto Research	[128]
Thimerosal		Sigma	sulfhydryl reagent; its oxidizing ability is related to the presence of mercury [124,129].
Diamide	****	Sigma	[125]
Hydroxyl radicals	**OH^−^** ^●^		[130]
Peroxynitrite	**ONOO^−^**		formed at a low rate by the reaction of NO^●^ with (O_2_^−^^●^) in a 1:1 stoichiometry for synthesis cf. [131].
Rose bengal	**4,5,6,7-tetrachloro-2',4',5',7'-tetraiodo fluorescein**	Sigma	Stain, clinical trials in some cancer therapies; generates singlet oxygen from triplet oxygen [132].
Normoxia			test solutions equilibrated with room air [126,133,134].
**Reduci** **ng agents**			
Chemical nomenclature	Abbrev./ Formula	Company	notes/literature
Dithiothreitol	**DTT**	Sigma	reduction of disulfide links [124,126,129,135].
β-mercaptoethanol	**β-ME**	Sigma	[125]
Glutathione	**GSH**		Physiological reduced form [136].
Nicotinamide adenine dinucleotide hydrate	**NADH**	Sigma	reduced form of physiological oxidized form NAD^+^ [137].
Thioredoxine	**TRX**	Sigma	in reduced form act as oxidoreductases [118].
Methionine sulfoxide reductase	**MSRA**	Sigma	converts Met-O to Met [109].
Hypoxia	****		bubbling of experimental solutions with nitrogen gas [126,133,134].
**Gasotransmitters**	****		
Nitric oxide, gas—donors	**NO**	Alexis	short lived radical (seconds) acts directly or via the sGC—cGMP—PKG signaling pathway [138]. NO range of (10^−9^ to 10^−7^ M) [124,132].
Diethylamine NONOate	**DEA-NO**	Corp.	
Sodium nitroprusside	**SNP**	Cayman	
S-Nitroso-*N*-Acetyl-d,l-Penicillamine, *etc*.	**SNAP**	Sigma	
Hydrogen sulfide, gas—donors	**H_2_S**	Sigma	[60,129,139]
Sodium hydrogen sulfide	**NaHS**		
Sodium sulfide, *etc*.	**Na_2_S**		
Carbon monoxide, gas—donors	**CO**	Sigma	[140]
Carbon monoxide releasing molecules	**CORM-A1**		[141,142]
	**CORM-2**		[143]
	**CORM-3**		[144]
*N*-ethylmaleimide	**NEM**	Sigma	alkylating agent [128]

Since these amino acids are particularly sensitive to redox reactions ROS may act on these targets in an autocrine or paracrine manner, *i.e*., on the cell itself or in its near vicinity. Other amino acids, such as arginine, lysine, proline, histidine, tryptophan and tyrosine may also provide targets for oxidation. The reactions may proceed extremely fast as with hydroxyl radicals (^•^OH) with a life-time of a few nanoseconds and a diffusion radius of a few nanometers [130], whereas the weaker radical nitric oxide (NO) has a life span of several seconds and a diffusion radius of several hundred micrometers [145]. Redox mechanisms linked to cell metabolism appear extremely important in the modulation of cellular signaling pathways and for the regulation of ion channels and electrical activity.

Human BK (Slo1) channels have 29 cysteines and 30 methionines per α-subunit which adds to 116 and 120 residues for each tetrameric complex [113]. Due to conformational properties many of these amino acids are buried within the 3D-structure of the protein and hence are not readily accessible to modifications. Functional consequences of oxidation of cysteine and methionine residues have been reviewed by Sahoo *et al.* (2014) [113].

## 4. Ion Channels and Oxidative Stress

Ion channels are modulated and regulated by a vast array of mechanisms, drugs and ligands. One of the first recognized and more detailed investigated posttranslational modifications of ion channels was their modulation by phosphorylation. In the human genome more than 500 putative kinase genes have been identified [146]. The attachment of phosphate groups to amino acids by protein kinases and the antagonistic action of phosphatases paved the way in our conceptualization of posttranslational modification of ion channel functioning [52,53,59,147,148,149]. Thereafter the field was open for more modulatory factors from signaling pathways, such as by G-proteins or second messengers, by redox mechanisms, by the action of gasotransmitters, such as nitric oxide (NO), carbon monoxide (CO), or hydrogen sulfide (H_2_S) [1,44,57,59,86,150] or more recently by S-acylation (the post-translational attachment of fatty acids to cysteine residues) [151]. Modulation of ion channels is involved in physiological processes such as transmitter release, hormone secretion or muscle contraction to name just a few [11,52,152,153,154,155]. Alterations of channels proteins by oxidative stress and its functional consequences constitutes one further way of channel modulation in a great variety of cells and tissues which will be covered in the following sections.

### 4.1. BK Channels and Redox Modification/Regulation

The brain, although it only accounts for 2% of the body weight, consumes ~20% of O_2_ and hence during metabolic activity produces large amounts ROS [156]. High amounts of O_2_ appear to be needed for the production of ATP which is used to maintain ion homeostasis in the brain in order to run active ion transport to establish transmembrane ion concentration gradients, intracellular neuronal transport systems or the synaptic transmission/neurosecretion machinery.

From brain-derived human BK channels (hslo) expressed in HEK cells (Human Embryonic Kidney cells) DiChiara and Reinhart (1997) [135] provided first evidence that the reducing agent dithiothreitol (DTT) shifted the voltage activation of the channels to more negative potentials and increased current activation as well as channel open probability. These modulations cause the channels to open earlier, *i.e*., during action potential repolarization and shorten the action potential duration which speeds up repetitive firing. Oxidation, by using hydrogen peroxide (H_2_O_2_), had the opposite effect. In contrast, BK channels cloned from *Drosophila* (dslo) were not modulated by DTT. It was suggested that in hslo proteins disulfide bonds were formed between cysteines—predominantly at the channels’ large interior C-terminus region. Soh *et al.* 2001 [136], reported that in neonatal rat hippocampal neurons the reducing reagent glutathione (GSH) increased BK channel activity whereas its oxidized form (GSSH) had the opposite effect indicating a redox modulatory mechanism when applied to the intracellular side of the cell membrane. On the other hand, after intracellular application of the oxidizing agent DTNB, Gong *et al.* (2000) [127] and Gao and Fung (2002) [157] reported an increase of open probability and decrease of closing times of BK channels from adult native hippocampal CA1 pyramidal neurons, while the reducing GSH had no apparent effect on the channel activity.

As natural reducing agent, the sulfhydryl specific reagent GSH is present in millimolar concentrations within cells. Depending on the cell type the intracellular concentration of GSH ranges between 1 mM and 10 mM whereof 98% exist in reduced form [158,159,160]. GSH rapidly (within 1 minute) in a concentration dependent manner increased BK channel activity in excised patches (a membrane patch which is removed from the cell like an inside out or outside out patch is called excised) of the cell when applied to the intracellular face of the membrane (of non-identified rat hippocampal cells) and this effect was rapidly reversible after wash-out [136]. In its oxidized form glutathione (GSSG) inhibited single channel activity which again was readily reversible. Hence, under physiological conditions GSH and GSSG form a redox pair where the ratio of GSH/GSH+GSSG appears responsible for the modulation of channel activity. Evidence suggests that GSH varies between brain regions and is augmented in glia cells compared to neurons [160,161,162] which makes neurons more susceptible to oxidative stress such as in Parkinson’s disease where GSH levels in neurons are decreased [163,164].

Mammalian neurons are highly vulnerable to oxygen (O_2_) deprivation. It has been hypothesized that the resulting depolarization of the membrane resting potential of these cells by hypoxia maybe in part mediated by inhibition of K^+^-currents. In neocortical neurons of mice hypoxia inhibited BK channel open probability in cell-attached patches but not in the excised inside-out mode [165]. Glutathione and application of DTT increased BK channel open probability. The experimental results suggested that O_2_ deprivation modulates BK channel activity by some cytosolic process(es) altering Ca^2+^ sensitivity resulting from intracellular pH changes or phosphorylation. These channels were, however, iberiotoxin and charybdotoxin insensitive which makes it questionable if these channels are genuine BK channels or if these channels were associated with β4 subunits which causes insensitivity to these toxins [65].

Lewis *et al.* (2002) [134] reported that the open probability of recombinant BK channel α-subunits from human brain, when co-expressed with β1 subunits in HEK cells in excised inside out patches, is oxygen-sensitive and reversibly suppressed by hypoxia. The experiments further indicated that the inhibition of channels by hypoxia was voltage-independent, reduced their Ca^2+^ sensitivity and did not require soluble intracellular factors.

Wyatt and Peers, (1995) [166] described O_2_-sensitive K^+^ currents in carotid body chemoreceptors cells of neonatal rats. In single channel and perforated patch whole cell recordings the authors reported on charybdotoxin sensitive channels which were inhibited by lowered PO_2_ and required cytosolic factors for normal functioning as well as O_2_ sensing. Hypoxia, anoxia or charybdotoxin depolarized the cells suggesting that closure of these channels leads to cell depolarization which is sufficient to activate voltage-gated Ca^2+^ channels and hence increased transmitter release. Jiang and Haddad (1994) [133] reported Ca^2+^ and voltage-dependent K^+^ channels of large conductance from rat central neurons in cell free, excised membrane patches, which were blocked by ATP. These channels were reversibly inhibited by hypoxia - but independent of cytosolic factors. Although ATP inhibition of BK channels has been reported [167] their identity as genuine BK channels remained unclear. Furthermore, hypoxia has also been reported to inhibit BK channel activity of rat carotid body type I cells in both whole cell and in excised single channel recording [126]. However, reduction of the channels using DTT increased, whereas oxidation using DTDP decreased channel open probability. From their experiments the authors concluded that hypoxic inhibition is not related to channel reduction and modulation of channels does not require cytoplasmic mediators. Interestingly, CO reverts hypoxic inhibition of the BK channels suggesting that CO binds to the channels or some hemoprotein sensor linked to the channels. In fact, such hemo-proteins have been also postulated for other preparations [168]. Oxidative modulation of K^+^ channels in the nervous system has been recently comprehensively summarized [113,117].

Perturbation of ion homeostasis is fundamentally involved in causing cell death after ischemia. Ca^2+^ excitotoxicity, which leads to the release of ROS, generates degrading enzymes or apoptotic factors [111,169]. One of the counteracting measures initiated by intracellular Ca^2+^ accumulation is the activation of BK channels. In excitable cells opening of BK channels causing hyperpolarization of cells provides a negative feedback for Ca^2+^ influx through voltage dependent Ca^2+^ channels and hence is involved in the regulation of action potential duration, neurotransmitter release or modulation of excitotoxicity [48,170,171,172]. Organotypic hippocampal slice cultures *in vitro* exposed to oxygen and glucose deprivation initiates cell death of CA1, and less of CA3 neurons. Treatment with the BK channel blockers, paxilline or iberiotoxin, increased this effect suggesting that BK channels act as a kind of “emergency brake” in ischemia [173]. Interestingly these treatments also increased the vulnerability of granula cells which are normally resistant to ischemic treatment. This raises the question as to the possible differences in the BK channel setting of these neurons. Accordingly, BK channels are considered as potential molecular targets for neuro-protective therapy in stroke. Using *in vivo* experimentation the issue was further investigated [174]. Focal ischemia (by cerebral artery occlusion) produced neuronal death in BK^−/−^ knock-out mice which was significantly increased compared to wild type animals. Organotypic hippocampal slice cultures if exposed to ischemia-like conditions experienced neuronal death in BK knock-out animals which was significantly increased compared to wild type cultures indicating that neuronal BK channels are important for protection against ischemic brain damage [174]. On the other hand, Kulawiak and Szewczyk (2012) [175] reported that inhibition of BK channels by the specific toxin paxilline dose dependently protected hippocampal neurons against glutamate induced cell death. However, iberiotoxin and charybdotoxin were not cytoprotective. From this and further data the authors concluded that the cytoprotective effect of paxilline was not dependent on BK channel inhibition. Although the experiments are not directly comparable to the studies of Liao’s group [174], since neuronal death was initiated by different procedures (low oxygen vs high glutamate) the results cause concern about the general mechanism.

Investigation of field excitatory postsynaptic potentials (fEPSPs) revealed that fEPSPs were depressed in an Alzheimer’s disease (AD) mouse model compared to age-matched controls [176]. BK channel blockers (charybdotoxin, paxilline) enhanced the fEPSP-potentials giving rise to the notion that impaired Ca^2+^ homeostasis and/or ROS generation may be considered as the underlying mechanisms.

Preconditioning, a phenomenon in which a non-harmful stress stimulus renders cells tolerant to a following otherwise damaging stimulus (see also below), has been tested in rat cortical neuronal cultures [177,178]. The BK channel opener NS1619, a potent inducer of delayed or immediate neuronal preconditioning, dose-dependently protected cells against toxic insults (oxygen/glucose deprivation, H_2_O_2_, or glutamate excitotoxicity) but protection was not blocked by BK channel inhibitors. Since NS1619 increases ROS generation, activates the phosphoinositide 3-kinase pathway and inhibits caspase activation it was proposed that it acts cytoprotective via these mechanisms rather than via BK channel activation. In immediate preconditioning modulation of NMDA receptors by ROS and up-regulation of superoxide dismutase activity followed by decreased Ca^2+^ influx, a reduction in oxidative stress upon glutamate exposure independent of BK channel activation was proposed [177].

Vertebrobasilar insufficiency (VBI) represents transient clinical symptoms including vertigo and hearing loss caused by decreased blood supply to the brain. Using whole cell patch clamp experiments at medial vestibular nucleus (MVN) neurons in brain slices Xie *et al*. (2014) [179] found that brief hypoxia elicits depolarization of the resting membrane potential and an increase of action potential discharge frequency. Furthermore, hypoxia decreased BK-mRNA levels as well as BK channel activity, the latter being alleviated by application of the BK channel opener NS1619. These experiments suggest a neuro-protective role of BK channels and a potential target for treatments in ischemia or stroke.

Hemin, an oxidation product of heme is released from decomposed erythrocytes and appears to play a role during hemorrhagic stroke or brain trauma (indicating intracerebral hemorrhage) [180]. Hemin inhibits BK channels of plasma and mitochondrial membranes [180]. The BK channel opener NS1619 attenuates ROS generation of brain mitochondria under conditions that allow for reverse electron flow [181], which may provide the reason for the different action of NS1619 on mitochondria compared to cells. The addition of hemin inhibits this effect by about 30% which is comparable to the specific BK channel blocker iberiotoxin [180]. Hence, the inhibition of mtBK channels by hemin may constitute a novel mechanism of neurotoxicity contributing to intracerebral hemorrhage. The authors suggest that an early anti-hemin therapy could help to prevent or diminish cytotoxicity [182].

There are no consistent results on the effects of oxidizing *vs.* reducing agents on BK channel activity. Although there is only one gene for BK channel expression many other factors can influence channel behavior, such as auxiliary subunits being present or absent, or differential splicing, as outlined in the previous section. Furthermore, different recording techniques using either whole cell recordings, where signaling pathways may interfere, *vs.* excised patch recordings, where channel activity is more directly accessed, may play a role. Also, the natures of the oxidizing/reducing compounds and their accessibility to the target site have to be considered.

### 4.2. BK Channels and Oxidative Stress at Muscle/Endothelial Cells

BK channel activity from excised inside-out tracheal myocytes was modified by sulfhydryl redox agents [125]. The reducing agent DTT augmented, whereas the oxidizing agent thimerosal inhibited channel open probability which persisted following wash-out of the drugs but were reversed by counteracting reagents. Alkylation of channel proteins to remove free thiols prevented the action of sulfhydryl altering agents. The experiments suggested that the inhibition by oxidizing compounds is caused by covalent modification of cytosolic channel thiol groups, likely cysteine residues. Glutathione (GSH) at low concentrations also significantly augmented BK channel activity indicating that alterations by the reducing GSH are of physiological relevance [125].

These results were in contrast to findings by other authors. In inside-out patches from isolated smooth muscle cells of rabbit pulmonary arteries, BK channel activity was increased by oxidizing agents such as nicotinamide adenine dinucleotide (NAD), 5'5-dithiobis(2-nitrobenzoic acid) (DTNB) or the oxidized form of glutathione (GSSG), whereas the reducing agents such as dithiothreitol (DTT), 2-hydroxy-1-ethanethiol (β-mercaptoethanol, (BME)), the reduced form of nicotinamide adenine dinucleotide (NADH) or GSH decreased channel activity [123]. Extracellular application of hydrogen peroxide (H_2_O_2_) relaxes porcine coronary arteries by increasing BK channel activity. The effect was observed in the cell attached mode and after excision of the patch to the inside-out mode and was mimicked by arachidonic acid suggesting the involvement of the lipoxygenase signaling pathway [120]. At the same preparation, Hayabuchi *et al*. (1998) [183] reported that the H_2_O_2_ initiated BK channel dependent vascular relaxation was mediated in part by direct action on the channels and in part by activation of the cGMP signaling pathway. From the experimental results, it appears feasible that both direct redox modulation at BK channel residues and/or indirect modulation occurs via some signaling pathway, which is initiated by redox processes. Explanations for these different findings may be the specificity of the preparations used, the expression of different auxiliary subunits with different redox sensitivity, the effect on the Ca^2+^ sensitivity directly or via auxiliary subunits *per se* or the preconditioned status of the channels [184].

Peroxynitrite (ONOO^−^) in biology can be produced from the reaction of the superoxide anion radical (O_2_**^−^**^•^) with the nitric oxide radical (NO^•^). In rat cerebral artery smooth muscle cells, ONOO^−^ has been found in whole cell BK current recordings as well as in inside out patches to decrease BK currents, BK channel open probability and channel mean open times, respectively [131]. The effects could be reversed by reduced GSH which also protected BK currents/channels from oxidation. The experimental results are consistent with ONOO^−^ being a contractile agonist of cerebral arteries and myocytes implicating a physiological mechanism in the modulation of vasoconstriction.

Infarct size in heart is profoundly reduced by ischemic preconditioning (IP)—a technique in which brief periods of ischemia precede sustained ischemia and provide tolerance to subsequent damaging insults. BK channels appear to be involved in this mechanism since pharmacological BK channel openers were found to be cardio-protective which was blunted by BK channel blockers, such as iberiotoxin or charybdotoxin [185,186,187] or after knock-out of the *KCNMA1* gene of neurons involved in the regulation of the heart beat [188].

ROS in addition to many other functions also appears involved in altering cellular ion homeostasis [189]. In heart ROS via oxidation of kinases (PKA, PKC, Calcium/Calmodulin Kinase II (CaMKII)) together with dysfunction of the sarcoplasmic reticulum can lead to perturbation of the Ca-homeostasis (which will indirectly affect BK channel activity) and finally cause heart failure.

### 4.3. Effects of Oxidative Stress on BK Channels of Epithelial Cells

In cultured alveolar epithelial A549 cells acute changes of oxygen tension (PO_2_) increased BK channel mean open time whereas chronic changes in PO_2_ did not affect expression, recruitment or function of BK channels or Na^+^ channel activity [190]. The authors concluded that BK channels serve as oxygen sensors. The mechanism of oxygen sensing was further elaborated by demonstrating that hemoxygenase-2 (HO-2), which is associated to BK channels enhances channel activity in normoxia [191]. It was found that carbon monoxide (CO), which is produced from O_2_ via HO-2, is a mediator of this function, indicating that HO-2 is an oxygen sensor.

In cultured human epithelial pigment cells, the oxidants, t-butyl hydroperoxide (t-BHP) or thimerosal suppressed BK outward currents recorded in whole cell configuration as well as BK channel open probability in the cell attached mode [192]. Internal application of ceramide prevented the oxidizing effect suggesting that BK channel inhibition may involve the intracellular generation of ceramide. In fact, ceramide can be produced in cells via the sphingomyelin pathway or by *de novo* synthesis [189,190]. H_2_O_2_ and other stressors have been reported to generate ceramide in various cells [193,194] and ceramide *per se* suppresses BK channel activity [195]. More information on this issue appears an interesting research endeavor.

It remained ambiguous for some time why oxidation in some cases increased, however, in others decreased channel activity. In their publication, Tang *et al*. (2001) [109] provided an explanation for the oxidative modulation of hSlo (human BK) channels considering the reversible oxidation of the amino acids cysteine and methionine. They showed that the agent chloramine-T (Ch-T), which oxidizes preferentially methionine to methionine sulfoxide (met(O)) or to met(O_2_), increased hSlo channel open probability. In contrast cysteine specific reagents, such as DNTB and other oxidizing agents decreased channel activity. Oxidation of these amino acids may also provide an explanation for the run-down of channels after patch excision (loss of channel activity with time), which exposes the channel inside to a more oxidized environment compared to the more reduced, normal intracellular milieu, and may also explain some earlier conflicting results. The authors were able to further provide evidence where at the channels the functional modification may take place. Internal channel blockers tetraethylammonium (TEA) or 1-methyl-4phenyl-1,2,3,6-tetrahydropyridine (MPTP) protected the channels from oxidation by Ch-T, indicating that residues oxidized by Ch-T are located near or within the channel pore and β-subunits are unlikely to contribute to this process. As outlined by the authors, oxidation of critical amino acids may be clinically relevant. In reperfusion after ischemic conditions the formation of free radicals may affect vascular BK channels. Methionine oxidation causing activation of BK channels may limit Ca^2+^ entry into cells and serve as neuronal protectant.

Tang *et al*. (2004) [61] further provided evidence that ROS to a large extent inhibited BK channels (α + β subunits expressed in HEK cells) by targeting cysteine residues near the intracellular Ca^2+^ binding site (Ca^2+^ bowl). In particular, a single oxidized cysteine residue, Cys911, prevented Ca^2+^ sensitivity. Further evidence that the α-subunit was the target for oxidation was obtained from experiments where its sole expression was sufficient for the suppression of currents. The authors line out that oxidative stress causing inhibition of BK channel activity and hence impairment of vascular relaxation and blood pressure may have crucial implications in disease and aging.

Auxiliary β-subunits also contain various amounts of cysteines (4–7) and methionines (3–11). Modifications at these amino acids are therefore amenable to affect channel activity. Zeng *et al.* (2003) [184] observed that reduction of extracellular disulfide linkages of the β3-subunit abolished current rectification and improved charybdotoxin blockade of the channels. The results indicate that the β-subunit appears to be close to the ion permeation pathway of the α-subunit and may regulate the gating mechanism and access of blocking molecules. Physiological consequences may be the dynamic regulation of channel inactivation by oxidative modification of SH groups.

The bovine β-subunit contains five cysteine residues which are conserved among various species such as rat, dog and humans [196]. Mutagenesis of each of the four cysteines present in the extracellular loop caused a profound reduction of charybdotoxin binding suggesting the generation of disulfide bridges between these residues. Methionines are only present at the intracellular N-terminus (3), and one is present at the end of the second transmembrane domain [197]. Oxidation of methionine in BK channel α-subunits (hSlo) by chloramine-T induced a leftward shift at the voltage axis to more negative values [109], *i.e*., the channels are activated already at more negative potentials closer to the membrane resting potential. In the presence of β1-subunits and under low intracellular Ca^2+^ conditions the hyperpolarizing shift was largely augmented, channel open probability increased and deactivation slowed [198]. However, this shift was independent of oxidation of methionine or cysteine residues of β1-subunits indicating that this auxiliary subunit has no direct effect but appeared to prime or to amplify the oxidation of the pore forming α-subunit residues. As a physiological implication it appeared that in electrically quiet cells at low baseline Ca^2+^ concentrations BK channels may have an increased impact on cellular electrical activity and hence influence vascular tone. In a following publication the authors demonstrated that the mutation of three conserved methionines located within the RCK domains (regulators of K^+^ conductance) at the cytoplasmic C-terminus eliminate oxidative sensitivity of the BK channels [199]. Oxidation of at least one of these key methionine residues was sufficient to increase the open probability of the channels and slowing of inactivation at low internal Ca^2+^. From theoretical considerations the authors proposed a mechanism of conformational changes in the gating ring structure which are transmitted to the voltage sensor domain. The functional effects caused by oxidation may serve as a protective mechanism by driving the cells into a more hyperpolarized, resting state and hence may be advantageous in pathological conditions such as after post-ischemic reperfusion or neurodegenerative disease to prevent neurotoxicity.

### 4.4. Oxidative Stress, BK Channels and Vascular Regulation

BK channels in the vascular system are modulated by agents naturally produced in the body, such as angiotensin II (Ang II), high glucose or arachidonic acid (AA) [200] which is modulated in diabetes by oxidative stress (ROS) [131,200,201]. In coronary smooth muscle cells BK channels are activated by arachidonic acid metabolites, in particular prostacyclin 2 (PGI2) [200]. In Zucker diabetic fatty rats channel activation is impaired caused by reduced production of prostacyclin 2 (PGI2) due to reduced PGI2 synthase. As a possible mechanism, ROS formation appeared to be involved. Lu *et al.* (2006) [201] reported that high glucose reduced BK channel density and channel kinetics by increasing ROS generation, in particular via H_2_O_2_-dependent oxidation of cysteine 911. In a further study it was shown that caveolae (small 50–100 nanometer invaginations of plasma membrane micro domains containing signaling components in close approximation) play an important role in mediating inhibition of BK channels by ROS [202]. In diabetic rat aortas, cav-1 expression (indicating caveolae) is upregulated and accompanied by an improved physical link between BK channels and the activated angiotensin-1 receptors (AT_1_R) signaling complex. Angiotensin II (Ang II)-activated AT1R receptors in diabetic rats stimulate enhanced non-phagocytic NAD(P)H oxidase and (NOX1) expression. This leads to an increased ROS-induced redox inhibition of BK channels and also to phosphorylation and nitration of tyrosine residues of the channels. The absence of caveolae (initiated by caveolin-1 (cav-1) knock-down or gene ablation) caused disaggregation of the Ang II-BK channel signaling cascade and preserved BK channel function in diabetes. The molecular mechanisms delineated so far indicate that the composition and spatial arrangement of the signaling system is important for proper functioning of coronary blood flow and maybe an interesting target for further pharmacological developments in the treatment of hypertension or atherosclerosis [202].

Another mechanism of BK channel activation of vascular tissue by H_2_O_2_ was presented by Burgoyne *et al*. (2007) [203] and Zhang *et al*. (2012) [204]. H_2_O_2_ which serves as an endothelium-derived hyperpolarizing factor (EDHF) causes dilation of human coronary arterioles by activation of BK channels. Cell-attached single channel recordings as well as inside out channel recordings from coronary smooth muscle BK channels revealed strong evidence that H_2_O_2_ increased channel open probability (Po) which requires intracellular signaling by a mechanism involving disulfide dimerization of the redox sensitive cGMP-dependent protein kinase (PKG-Iα). Hence the H_2_O_2_ mechanism of action appears different to the NO mediated sGC-stimulated cGMP-PKG pathway but acts downstream directly at the PKG-Iα level. Release of H_2_O_2_ from endothelial cells appears to play a prominent role as endogenous regulator under normal conditions and in diseased states [204].

There is also evidence that anoxia may act via protein kinase C (PKC). In Western painted turtle cortical pyramidal neurons anoxia reversibly reduced the open probability of BK channels in the cell-attached mode but not in excised patches [205]. The PKC inhibitor chelerythrine prevented the anoxic effect whereas the PKC activating phorbol ester PMA (phorbol-12-myristate-13-acetate) decreased Po during normoxia. It was concluded that the inhibition of BK channels activity prevents K^+^ efflux, preserves K^+^ homeostasis and hence reduces cellular ATP usage and promotes survival of neurons which enables the animals to adapt to low oxygen levels. Similarly, an anoxia dependent inhibition of Na^+^ currents recorded from rat hippocampal neurons via a PKC dependent mechanism has been reported previously [206].

In cat cerebral arterial muscle cells, hypoxia superfusion produced a transient increase in mean open time of BK channels in excised inside-out patches and this effect was independent of changes of the internal Ca^2+^ concentration or pHi [207]. Furthermore, reduced PO_2_ caused dilation of cerebral arterial segments which was attenuated by tetraethylammonium (TEA). In a following publication Gembremedhin *et al.* (2008) [208] reported that in rat cerebral arterial muscle cells hypoxia reversibly enhanced BK channel open-state probability in cell-attached patches but this effect was absent in either excised inside-out or outside-out patches. The results further indicated that hypoxia induced the generation of superoxide which caused a reduction in endogenous level of 20-hydroxyeicosatetraenoic acid (20-HETE) that may account for the hypoxia-induced activation of arterial BK channel currents and cerebral vasodilatation.

BK channels, among other K channels, are associated with decreased uterine vascular tone and an increase of uterine blood flow during pregnancy in order to optimize adequate nutritional supply and tissue oxygenation for the fetus. Chronic hypoxia during gestation by suppression of BK channel function increases uterine vascular tone and decreases uterine blood supply which increases the risk of preeclampsia and retarded fetal growth [209,210,211]. In addition, expression of BK channels during pregnancy is upregulated but down-regulated during chronic hypoxia. At the same time, protein kinase C (PKC) activity is increased during hypoxia which inhibits BK channels, whereas under normoxia these effects are reversed [212].

Sex steroid hormones (estrogens) regulate uterine vascular tone and uterine blood flow by increasing BK channel density (in particular β1-subunits) and activity, whereby also PKC signaling appears an important modulatory mechanism. In contrast, progesterone appears to inhibit BK channels [213] but its contribution to regulating uterine vascular tone needs further investigation. Exposure to hypoxia during pregnancy attenuates the effects of sex steroid hormones/receptors, leading to enhanced PKC activation resulting in the inhibition of BK channel activity and increased pressure-dependent myogenic tone in pregnant uterine arteries [211]. Furthermore, hypoxia-mediated ROS activation during gestation inhibits steroid hormone mediated up-regulation of BK channel activity which may contribute to malfunctions during gestational hypoxia [214]. The evidence suggests that modulation of K^+^ channel activity during pregnancy conveys an important mechanism underlying hypoxia induced uterine vascular dysfunction [215].

### 4.5. Mitochondrial BK Channels (mtBK) and Oxygen

Mitochondria are a major source of ROS generation targeting BK channels [122,216,217,218]. The inner membrane of mitochondria contains BK channels (mtBK) [219,220,221] which appear essential in the production of ROS. mtBK channels appear to be inserted into the mitochondrial membrane with the toxin binding sites for charybdotoxin and iberiotoxin exposed to the mitochondrial intermembrane space (accessed by using outside-out patch configuration of the inner mitochondrial membrane). Consequently the C-terminal tail domain including the Ca^2+^ binding site is localized to the mitochondrial matrix. mtBK channels of cardiac tissue have recently been specified as being encoded by the *KCNMA1* gene and hence appear to be identified as genuine BK channels [222].

Opening of BK channels allows K^+^, which is present in a high concentration in the cytosol, to flow into the negatively charged mitochondrial matrix and depolarize the organelle (reviewed in [216]). This reduces the driving force for Ca^2+^ and hence Ca^2+^ influx which reduces Ca^2+^ overload of mitochondria. In fact, mtBK channel activation and K^+^ uptake was reported to confer cytoprotection to heart infarction [221]. Further studies revealed that preconditioned activation of mtBK channels by the channel opener NS-1619 or NS11021 reduces superoxide production and reduces Ca^2+^ overload which improved Ca^2+^ homeostasis and redox state after ischemia/reperfusion in isolated guinea pig hearts [122,223,224,225]. Another mtBK channel opener 12,14-dichlorodehydroabietic acid (diCl-DHAA) was also reported to reduce ischemic injury in rat cardiac myocytes [226]. The steroid, 17β-estradiol, enhances the activity of cardiac mtBK channels, but only in the presence of the auxiliary β1-subunit and increased survival of myocytes under simulated ischemia [227]. In addition the β1-subunit appeared to interact with the cytochrome c oxidase subunit I. These findings may help to improve ischemic diseases such as heart attack in postmenopausal women by applying an estrogen-induced cardio-protective treatment. Furthermore, opening of mtBK channels of brain-derived mitochondria appears to inhibit ROS production suggesting that this may also contribute to the beneficial effects of BK channel openers on neuronal survival [175].

In the heart, BK channels appear absent from the sarcolemma, but the channels are present in mitochondrial membranes (reviewed in [97,228]). The opening of BK channels by hypoxia exerts a cytoprotective effect, which has been attributed to the mechanism mentioned above. In addition β1-subunits are highly expressed together with mtBK α-subunits that contain two *N*-glycosylation sites at their C-terminus which on enzymatic deglycosylation cause activation of BK channels [229,230]. Borchert *et al*. (2013) [231] reported that the BK channel opener NS11021 or H_2_O_2_ increased survival of cardiomyocytes in simulated ischemia/reperfusion experiments. The cytoprotective effect was abolished by the specific BK channel inhibitor paxilline or tempol, a specific antioxidant. The study indicates that the mechanism for this protection requires ROS signaling suggesting that activation of mtBK channels protect the cells against injury. Experiments using chronic hypoxic rats exposed to brief intermittent re-oxygenation, attenuated cardio protection possibly by a mechanism involving oxidative stress and suppression of mtBK activity [232].

Mitochondrial BK channels (mtBK) derived from streptozotocin-induced diabetic rat brain incorporated into lipid bilayer membranes were found to exhibit a decreased open probability and conductance [233]. In addition, both BK α- and β4 subunits expression was down-regulated. This evidence and an increased production of ROS during diabetic conditions, as proposed by others [201], were suggested to account for the abnormalities in channel gating. In the studies on molecular level, Lu *et al*. (2006) have shown that C911 is a major molecular target at the channel protein for the redox modulation by high glucose [201]. This body of acquired knowledge may be useful for the development of improved treatment of diabetics.

### 4.6. Gasotransmitters and ROS Effects on BK Channels

In this review, we only briefly outline some aspects of the interaction of BK channels and gasotransmitters. Recent information on this issue has been summarized by [63,117,234]. Nitric oxide (NO) produced by endothelial NO synthase (eNOS) appears to prevent atherosclerosis by interaction with the vascular NO generating system [235]. NADPH oxidases are a major source of ROS leading to various types of vascular pathophysiology. In atherosclerosis bioactivity of NO is decreased by reduced NO synthesis due to an increased NO inactivation.

Endothelial cells by appropriate stimulation (humoral or hemodynamic), release vasodilatory factors, such as NO, whereas vasoconstrictory ROS are generated as byproducts of oxygen metabolism [121]. Single BK channels recorded from *in situ* renal artery endothelium preparations revealed that NO increased channel open probability by two mechanisms: either by direct action on the channel proteins (probably by nitrosylation) or by indirect action via cGMP (mediated by phosphorylation) [132]. In contrast, ROS (H_2_O_2_ *etc.*) resulted in irreversible channel inactivation. The authors concluded that BK activation by NO creates a positive feedback by autocrine regulation of endothelial function, whereas ROS inhibits BK channels which impairs hyperpolarization of endothelial cells and consequently prevents vasodilation.

NO inhibits M-currents of rat sensory neurons from trigeminal ganglia [236]. M-type K^+^ channels are subthreshold voltage-gated K^+^ channels of the six TM type (designated Kv7, *KCNQ* gene family) which are inhibited by M1 muscarinic acetylcholine receptors and affect excitability of neurons in the central and peripheral nervous system or cardiac tissue. The authors identified a site of NO action within the cytosolic channel linker between transmembrane domains 2 and 3, which appears also to be a site of oxidative modification by ROS. However, NO and oxidative modifications exhibit opposing effects on M-currents. These channels therefore appear to contain a dynamic redox sensor that is responsible for dynamic M-current modulation by gasotransmitters and ROS and may play a role in trigeminal disorders such as headache and migraine. It appears interesting to probe this channel site for different types of gasotransmitters and ROS also at BK channels. Other types of K^+^ channels and oxidative stress are discussed in [112,117,237,238].

In our experiments using GH3 pituitary tumor cells, sodium hydrogen sulfide (NaHS), a H_2_S donor, increased channel open probability which was prevented by the reducing agent DTT, whereas the oxidizing agent thimerosal increased channel open probability in the presence of H_2_S [129]. The effect was linked to the reducing action of H_2_S on sulfhydryl groups of the channel protein. This finding is in concert with a report by Liu *et al*. 2009 [239] indicating that the H_2_S donor NaHS prevents postischemic mitochondrial dysfunction by a BK channel dependent mechanism. The development of drugs interfering with H_2_S signaling might be rewarding in the treatment of mitochondrial linked diseases or to BK channel dependent high blood pressure. Some reagents used to study the modulation of BK channels by gasotransmitters are listed in Table 1.

### 4.7. Oxidative Stress, Proliferation and Various Types of K^+^ Channels

In pulmonary artery smooth muscle cells various types of K^+^ channels, such as K_V_, K_ir_, BK, K_2P_, participate in vascular remodeling (proliferation, apoptosis) [240]. In general, loss or inhibition of K^+^ channel function contributes to pulmonary pathogenesis which leads to a decrease of proliferation and inhibition of apoptosis. Hypoxic inhibition of ROS production causing inhibition of K^+^ channels leads to depolarization, opening of Ca^2+^ channels and augmentation of intracellular Ca^2+^ which finally results in vasoconstriction of small pulmonary arteries. K^+^ channel expression is transcriptionally regulated and cells need K^+^ channels to proceed through the cell cycle (in particular G1 progression) [240]. Hence, K^+^ channels are appealing therapeutic targets in pulmonary arterial hypertension. Oxidized low-density lipoprotein lysophosphatidylcholine (LPC) increases BK channel open state probability by causing capacitative Ca^2+^ influx [241]. Ca^2+^ accumulation increases ROS production that causes reduction of NO generation which promotes proliferation of cultured human endothelial cells. In addition LPC-induced BK activation contributes to increased cGMP levels, if ROS production was prevented by transfection with antisense oligonucleotides against NAD(P)H oxidase. Cancer incidence also appears associated with a great variety of ion channels, in particular K^+^ channelopathies [242,243,244]. In fact, almost all known types of K^+^ channels have been implicated in oncogenic processes. The dysregulation of K^+^ channel expression (mostly overexpression) and resulting dysfunction of K^+^ channels correlates with dysregulation of proliferation, malignant growth and migration (metastatic spread) of tumorigenic cells. As a general phenomenon, the membrane potential of cancer cells is more depolarized [245] and the resting membrane potential oscillates during the cell cycle, being more depolarized during S and G2 phases [246,247].

Polyamines (mainly putrescine, spermidine and spermine) exhibit a wide array of functions from modulating ion channels, involvement in apoptosis, carcinogenicity, cell proliferation or development. Modulatory properties of polyamines concerning K^+^ channels involved in cell proliferation has been summarized by Weiger and Hermann (2014) [248]. Here we briefly comment on some issues in the context of oxygen impact. Polyamine deficient yeast (*Saccharomyces cerevisiae*), for example, is very sensitive to oxygen. Polyamine depleted cells accumulate ROS, develop an apoptotic phenotype and die after incubation in polyamine-deficient medium [249]. Addition of spermine caused a marked decrease in ROS accumulation and some protection against cell death. The data indicate that part of the function of polyamines is protection of the cells from accumulation of ROS.

Polyamine biosynthesis is increased during cerebral ischemia through induction of ornithine decarboxylase (ODC), a key enzyme for their synthesis. Inhibition of ODC prevents ischemic brain injury [250]. Metabolization of polyamines by polyamine oxidases generates cytotoxic aldehydes and ROS. Polyamines may therefore constitute crucial players in oxidative stress. There is little information on polyamines, ion channels (BK) and oxidative stress which remains an interesting field for future investigations.

### 4.8. Oxidative Stress of BK Channels in Tumors

Tumors are remarkably tolerant to hypoxia whereas normal nerve cells have a high demand on oxygen and rapidly die on hypoxia. This of course raises the question concerning the mechanisms involved. Evidence suggests that the modulation of ion channels and intracellular signaling pathways may be a key in determining cellular tolerance to low oxidative stress [251]. Expression of BK channels is increased in cancerous compared to healthy cells and correlates with the malignancy of the tumors [252,253]. It was hypothesized that differences in the expression of BK channels in tumor cells compared to healthy cells could be the reason for the differences in their response to hypoxia. These findings may provide an approach for novel vistas in cancer therapy.

Hypoxia in a variety of healthy cells in general decreases open probability of BK channels in the plasma membrane (plBK), whereas mtBK channels are opened by hypoxia [254,255]. Studies of normoxic *vs.* hypoxic conditions of single mtBK and plBK channels from human glioma cells showed that plBK channels were insensitive to hypoxia whereas mtBK channels open probability was increased during hypoxia [254,256]. Activation of mtBK channels by specific agonists had no effect on cell viability. In contrast, activation of plBK channels by other specific agonists, impaired cell viability of tumor cells [255,257] and this effect was increased by hypoxia [255]. It was suggested that the mechanism leading to cell death/apoptosis in normoxia is based on Ca^2+^ toxicity, *i.e*., it is mediated by an increase of cytosolic Ca^2+^ and activation of calpains [251,258]. The effect of hypoxia on mtBK channels appears to have different consequences on cell viability [254]. A cytoprotective effect has been attributed to (a) increased matrix K^+^, (b) prevention of Ca^2+^ overload, and c) closing of the mPTP pore. Mitochondrial permeability transition pores (mPTP) close on hypoxia. mPTP in the inner mitochondrial membrane are considered to activate a pathway for the release of pro-apoptotic factors from mitochondria (for further information about this topic the reader is referred to [259]).

### 4.9. Clinical Relevance

Oxidative modulation of BK channels is important not only in order to understand and treat diseased conditions like myocardial infarction or stroke but, as it recently came also into focus, for transplantation surgery. For instance, in the case of lung transplantation the graft is subjected to ischemia followed by reperfusion during routine procedure. During ischemia a number of cellular processes eventually lead to membrane depolarization and ROS generation [260] which in turn favors inflammation and cell death [261]. Noda *et al*. 2014 [262] found that preconditioning lung grafts with inhaled hydrogen, a cytoprotective gaseous signaling molecule, reduced the proinflammatory changes and led to better post-transplant graft function. The molecular mechanism behind this effect is, among others, a stimulation of hemeoxygenase-1 (HO-1) expression by hydrogen [262]. As already discussed above HO-1 activity results in production of CO which then activates BK channels [263]. Active BK channels will cause repolarization of the membrane resting potential and this way may contribute to improved lung graft transplantation. These discoveries again underline the importance and protective role of BK channels. In conclusion, ROS modulation may exert conformational alterations at the channel proteins with two major implications: (1) impairment or dysfunction of channels which may lead to diseases or death, such as in vascular impairment or heart attack, or (2) modifications at channels may lead to physiological modulation of channel functioning, such as in sensing of oxygen tension.

### 4.10. Perspectives

In a recent publication the authors report that a point mutation of a phenylalanine at the F380 position in the S6 transmembrane helix of BK channels greatly obviates channel opening [264]. Based on further functional experiments and molecular dynamic simulations they proposed a model where in the process of channel conduction a hydrophobic ring structure forms that acts as an integration node which affects the interaction between the voltage sensor and the pore. It appears interesting to probe this site for ROS manipulation.

Sulcatone (6-Methyl-5-hepten-2-one (C8H14O)), a prominent volatile component of human body odor that is less abundant in non-human animals, appears to specifically attract some species of female mosquitos (*Aedes aegypti*) to feed on our blood [265]. A prokaryotic protein with features reminiscent to an ancestral single domain bacterial K^+^ channel protein may have provided the evolutionary blueprint for pentameric ligand-gated ion channels which serve as odorant receptors (*Ore4*) for sulcatone [266]. Interestingly, sulcatone belongs to the family of oxidoreductases which are known to act on ion channels. It may be speculated that sulcatone acting on K^+^ channels which have been found to alter cellular signaling and are involved in controlling proliferation [248] may be interesting targets for future investigations.

Various types of other K^+^ channels, other than BK channels, such as voltage gated K^+^ channels (K_V_), ATP-gated K^+^ channels (K_ATP_) or (K_ir_), appear to be involved in vasomodulation [267,268]. Only a few other K^+^ channel studies in the context of oxidative stress, from which we may draw interesting information and extend our view, will be covered in this section. In a recent study, Park *et al*. (2015) [269] report that H_2_O_2_ relaxes rat mesenteric arteries which was reversed by application of the reducing agent dithiothreitol. The vasodilatory effect of H_2_O_2_ was reduced by the voltage gated K^+^ channels (K_V_) blocker 4-aminopyridine (4-AP) but was resistant to BK and inward rectifier K^+^ channels (K_ir_) channel blockers. Whole cell patch clamp studies further showed that K_V_ currents recorded from mesenteric smooth muscle cells were dose-dependently increased by H_2_O_2_ as well as by oxidized glutathione (GSSH) and prevented by glutathione reductase. Further studies showed that reduced glutathione (GSH) is incorporated into the K_V_ channel protein indicating S-glutathionylation of the channels by H_2_O_2_. Park *et al*. (2015) [269] now report that by an increased basal level of H_2_O_2_, *i.e*., under conditions of persistent oxidative stress, Kv channels were not activated, but rather inhibited by the addition of H_2_O_2_. The findings suggest that the actual cellular redox status affects S-glutathionylation of the K_V_ channels and determines the response of these K_V_ channels to H_2_O_2_.

Oxidative stress is also a hallmark of vascular disorders such as diabetic retinopathy. In this case ATP-sensitive K channels (K_ATP_) activity were found to be increased during exposure of retinal capillaries to H_2_O_2_ [270]. The effect on K_ATP_ was boosted by increasing the influx of Ca^2+^ into microvascular cells and oxidant-induced activation of Ca^2+^-permeable nonspecific cation channels. Furthermore, it was found that inhibition of K_ATP_ channels by the specific K_ATP_ blocker glibenclamide significantly lessened H_2_O_2_ induced microvascular cell death. The findings were suggested to provide new targets for pharmacological treatments of retinal microvasculature during oxidative stress.

## 5. Conclusions

In conclusion, the overall status of the present studies indicates that redox modifications of cysteines/methionines cause conformational alterations at the BK channel protein which translate into modulation of channel pore openings (gating) in which interference with the Ca^2+^ activation mechanism also appears to play a major role. If the target alterations occur at single sites or in combination, whether other amino acid targets bear functional relevance and how these manipulations affect conformational changes of the 3D-protein structure needs further investigation. Primary effects, directly at the channels, and/or secondary effects on signaling pathways, may be both relevant, but have to be clearly separated. BK channel/ROS modulation will certainly be an important target for development of pharmacological agents which function as channel openers/blockers opposing negatively effective redox mechanisms.

## Abbreviations

AA: Arachidonic acid
ACA: Acetaldehyde
Ach: Acetylcholine
AD: Alzheimer’s disease
Ang: II angiotensin II
4-AP: 4-aminopyridine
ATP: Adenosine triphosphate
AT_1_R: Angiotensin-1 receptor
β-ME: Mercaptoethanol
BME: 2-hydroxy-1-ethanethiol (β-mercaptoethanol)
BK: Big or maxi calcium-activated potassium channel
BK^−/−^: Knock-out animal
CaMKII: Calcium/Calmodulin Kinase II
cAMP: Cyclic adenosinemonophosphate
cav-1: Caveolin-1
cGMP: Cyclic gCanosinemonophosphate
CBS: Cystathionine b-synthase
Ch-T: Chloramine-T
CO: Carbon monoxide
CORM: Carbon monoxide releasing molecule
CREB: Cyclic AMP response element-binding protein
CSE: Cystathionine c-lyase
Cys or C: Cysteine
diCl-DHAA: 12,14-dichlorodehydroabietic acid
DTT: 1,4-dithio-DL-threitol (short: dithiothretiol)
DTDP: 2,2'-dithiodipyridine or 4,4'-dithiodipyridine
DTNB: 5,5'-dithiobis(2-nitrobenzoic acid)
EDHF: Endothelium-derived hyperpolarizing factor
EET: Epoxyeicosatrienoic acid
ER: Estrogen receptor
EtOH: Ethanol
fEPSP: Field excitatory postsynaptic potential
G-protein: Guanosine triphosphate (GTP) binding protein
GSH: Glutathione-reduced form
GSSH: Glutathione-oxidized form
20-HETE: 20-hydroxyeicosatetraenoic acid
H_2_O_2_: Hydrogen peroxide
H_2_S: Hydrogen sulfide
HEK: Human Embryonic Kidney cells
HO: Heme oxygenase
HO-2: Hemoxygenase-2
HS: Hydrogen sulfide anion
hSlo: Human BK channel
IK: Intermediate conductance K^+^ channel
IP: Ischemic preconditioning
K_ATP_: Adenosine-triphosphate dependent K^+^ channel
LPC: Lysophosphatidylcholine
LTP: Long-term potentiation
Met or M: Methionine
Met-O: Methionine sulfoxide
mM: Milli Molar
MPTP: 1-methyl-4phenyl-1,2,3,6-tetrahydropyridine
MSRA: Methionine sulfoxide reductases
mtBK: Mitochondrial BK channel
MTSEA: Methanethiosulfonate ethylammonium
MVN: Medial vestibular nucleus
NAD: Nicotinamide adenine dinucleotide
NADH: Nicotinamide adenine dinucleotide
NaHS: Sodium hydrogen sulfide
nBK: Nuclear BK channel
NCS: *N*-chlorosuccinimid
NO: Nitric oxide
NOS: NO synthase
nM: Nano Molar
NMDA: *N*-methyl d-aspartate
O_2_: Oxygen
O_2_**^−^**^•^: Superoxide anion radical
•OH: Hydroxyl radical
ONOO^−^: Peroxynitrite
ODC: Ornithine decarboxylase
P: Pore loop
PDE: Phosphodiesterase
PGI2: Prostacyclin 2 (Prostaglandin I_2_)
PKA, PKC, PKG: Protein kinase A, C, G
plBK: Plasma membrane BK channel
PMA: Phorbol-12-myristate-13-acetate
PO_2_: Oxygen tension
Po (Popen): Open probability of channels
PP2A: Protein phosphatase
PS: Phosphatidylserine
pS: Piko Siemens
PTP: permeability transition pore
RCK: Regulatory domain of K^+^ conductance
ROS: Reactive oxygen species
SK: Small conductance K^+^ channel
sGC: Soluble guanylyl cyclase
Slob: Slo binding protein
STREX: Stress-axis regulated exon
T: Transmembrane
t-BHP: t-butyl hydroperoxide
TEA: tetraethylammonium
TRX: Thioredoxine
VBI: Vertebrobasilar insufficiency

## References

[1] Hille B. (2001). Ion Channels of Excitable Membranes.

[2] Hodgkin A.L., Huxley A.F., Katz B. (1952). Measurements of current-voltage relations in the membrane of the giant axon of Loligo. J. Physiol..

[3] Hamill O.P., Marty A., Neher E., Sakmann B., Sigworth F.J. (1981). Improved patch-clamp techniques for high-resolution current recording from cells and cell-free membrane patches. Pflüg. Arch..

[4] Doyle D.A., Cabral J.M., Pfuetzner R.A., Kuo A., Gulbis J.M., Cohen S.L., Chait B.T., MacKinnon R. (1998). The structure of the potassium channel: Molecular basis of K^+^ conduction and selectivity. Science.

[5] Brueggemann L.I., Gentile S., Byron K.L. (2013). Social networking among voltage-activated potassium channels. Prog. Mol. Biol. Transl. Sci..

[6] Levitan I.B. (2006). Signaling protein complexes associated with neuronal ion channels. Nat. Neurosci..

[7] Armstrong C.M., Hille B. (1998). Voltage-gated ion channels and electrical excitability. Neuron.

[8] Yu F.H., Yarov-Yarovoy V., Gutman G.A., Catterall W.A. (2005). Overview of molecular relationships in the voltage-gated ion channel superfamily. Pharmacol. Rev..

[9] Bezanilla F. (2005). Voltage-gated ion channels. IEEE Trans. Nanobioscience.

[10] Long S.B., Tao X., Campbell E.B., MacKinnon R. (2007). Atomic structure of a voltage-dependent K^+^ channel in a lipid membrane-like environment. Nature.

[11] Dai S., Hall D.D., Hell J.W. (2009). Supramolecular assemblies and localized regulation of voltage-gated ion channels. Physiol. Rev..

[12] Catterall W.A. (1995). Structure and function of voltage-gated ion channels. Annu. Rev. Biochem..

[13] Catterall W.A. (2014). Structure and function of voltage-gated sodium channels at atomic resolution. Exp. Physiol..

[14] Catterall W.A. (2011). Voltage-gated calcium channels. Cold Spring Harb. Perspect. Biol..

[15] González C., Baez-Nieto D., Valencia I., Oyarzún I., Rojas P., Naranjo D., Latorre R. (2012). K^+^ channels: Function-structural overview. Compr. Physiol..

[16] Stölting G., Fischer M., Fahlke C. (2014). CLC channel function and dysfunction in health and disease. Front. Physiol..

[17] Gardos G. (1958). The function of calcium in the potassium permeability of human erythrocytes. Biochim. Biophys. Acta.

[18] Wei A.D., Gutman G.A., Aldrich R., Chandy K.G., Grissmer S., Wulff H. (2005). International union of pharmacology. LII. Nomenclature and molecular relationships of calcium-activated potassium channels. Pharmacol. Rev..

[19] Ghatta S., Nimmagadda D., Xu X., O’Rourke S.T. (2006). Large-conductance, calcium-activated potassium channels: Structural and functional implications. Pharmacol. Ther..

[20] Shipston M.J. (2014). S-acylation dependent post-translational cross-talk regulates large conductance calcium- and voltage- activated potassium (BK) channels. Membr. Physiol. Membr. Biophys..

[21] Salkoff L., Butler A., Ferreira G., Santi C., Wei A. (2006). High-conductance potassium channels of the SLO family. Nat. Rev. Neurosci..

[22] Cui J., Yang H., Lee U.S. (2009). Molecular mechanisms of BK channel activation. Cell. Mol. Life Sci..

[23] Berkefeld H., Fakler B., Schulte U. (2010). Ca^2+^-activated K^+^ channels: From protein complexes to function. Physiol. Rev..

[24] Cui J. (2010). BK-type calcium-activated potassium channels: Coupling of metal ions and voltage sensing. J. Physiol..

[25] Lee U.S., Cui J. (2010). BK channel activation: Structural and functional insights. Trends Neurosci..

[26] Grimm P.R., Sansom S.C. (2010). BK channels and a new form of hypertension. Kidney Int..

[27] Hill M.A., Yang Y., Ella S.R., Davis M.J., Braun A.P. (2010). Large conductance, Ca^2+^-activated K^+^ channels (BKCa) and arteriolar myogenic signaling. FEBS Lett..

[28] Wu Y., Yang Y., Ye S., Jiang Y. (2010). Structure of the gating ring from the human large-conductance Ca^2+^-gated K^+^ channel. Nature.

[29] Latorre R., Morera F.J., Zaelzer C. (2010). Allosteric interactions and the modular nature of the voltage- and Ca^2+^-activated (BK) channel. J. Physiol..

[30] Hermann A., Sitdikova G.F., Weiger T.M., Shad Kaneez F. (2012). BK Channels—Focus on Polyamines, Ethanol/Acetaldehyde and Hydrogen Sulfide (H2S). Patch Clamp Technique.

[31] Rothberg B.S. (2012). The BK channel: A vital link between cellular calcium and electrical signaling. Protein Cell.

[32] N’Gouemo P. (2011). Targeting BK (big potassium) channels in epilepsy. Expert Opin. Ther. Targets.

[33] Hoshi T., Pantazis A., Olcese R. (2013). Transduction of voltage and Ca^2+^ signals by Slo1 BK channels. Physiology.

[34] Toro L., Li M., Zhang Z., Singh H., Wu Y., Stefani E. (2014). MaxiK channel and cell signalling. Pflüg. Arch..

[35] Ge L., Hoa N.T., Wilson Z., Arismendi-Morillo G., Kong X.-T., Tajhya R.B., Beeton C., Jadus M.R. (2014). Big Potassium (BK) ion channels in biology, disease and possible targets for cancer immunotherapy. Int. Immunopharmacol..

[36] Kyle B.D., Braun A.P. (2014). The regulation of BK channel activity by pre- and post-translational modifications. Front. Physiol..

[37] Yang H., Zhang G., Cui J. (2015). BK channels: Multiple sensors, one activation gate. Front. Physiol..

[38] Fodor A.A., Aldrich R.W. (2009). Convergent evolution of alternative splices at domain boundaries of the BK channel. Annu. Rev. Physiol..

[39] Xie J., McCobb D.P. (1998). Control of alternative splicing of potassium channels by stress hormones. Science.

[40] Erxleben C., Everhart A.L., Romeo C., Florance H., Bauer M.B., Alcorta D.A., Rossie S., Shipston M.J., Armstrong D.L. (2002). Interacting effects of N-terminal variation and strex exon splicing on slo potassium channel regulation by calcium, phosphorylation, and oxidation. J. Biol. Chem..

[41] Berthois Y., Katzenellenbogen J.A., Katzenellenbogen B.S. (1986). Phenol red in tissue culture media is a weak estrogen: Implications concerning the study of estrogen-responsive cells in culture. Proc. Natl. Acad. Sci. USA.

[42] Holdiman A.J., Fergus D.J., England S.K. (2002). 17β-Estradiol upregulates distinct maxi-K channel transcripts in mouse uterus. Mol. Cell. Endocrinol..

[43] Norfleet A.M., Thomas M.L., Gametchu B., Watson C.S. (1999). Estrogen receptor-alpha detected on the plasma membrane of aldehyde-fixed GH3/B6/F10 rat pituitary tumor cells by enzyme-linked immunocytochemistry. Endocrinology.

[44] Hermann A., Sitdikova G.F., Weiger T.M., Hermann A., Sitdikova G.F., Weiger T.M. (2012). Modulated by Gasotransmitters: BK Channels. Gasotransmitters: Physiology and Pathophysiology.

[45] Hermann A., Gorman A.L. (1979). External and internal effects of tetraethylammonium on voltage-dependent and Ca-dependent K^+^ currents components in molluscan pacemaker neurons. Neurosci. Lett..

[46] Lang D.G., Ritchie A.K. (1990). Tetraethylammonium blockade of apamin-sensitive and insensitive Ca^2+^-activated K^+^ channels in a pituitary cell line. J. Physiol..

[47] Zhou Y., Lingle C.J. (2014). Paxilline inhibits BK channels by an almost exclusively closed-channel block mechanism. J. Gen. Physiol..

[48] Gribkoff V.K., Lum-Ragan J.T., Boissard C.G., Post-Munson D.J., Meanwell N.A., Starrett J.E., Kozlowski E.S., Romine J.L., Trojnacki J.T., Mckay M.C. (1996). Effects of channel modulators on cloned large-conductance calcium-activated potassium channels. Mol. Pharmacol..

[49] Thompson J., Begenisich T. (2009). Mechanistic details of BK channel inhibition by the intermediate conductance, Ca^2+^-activated K channel. Channels.

[50] Gola M., Crest M. (1993). Colocalization of active KCa channels and Ca^2+^ channels within Ca^2+^ domains in helix neurons. Neuron.

[51] Issa N.P., Hudspeth A.J. (1994). Clustering of Ca^2+^ channels and Ca^2+^-activated K^+^ channels at fluorescently labeled presynaptic active zones of hair cells. Proc. Natl. Acad. Sci. USA.

[52] Levitan I.B. (1994). Modulation of ion channels by protein phosphorylation and dephosphorylation. Annu. Rev. Physiol..

[53] Levitan I.B. (1999). Modulation of ion channels by protein phosphorylation. How the brain works. Adv. Second Messenger Phosphoprotein Res..

[54] Tian L., Duncan R.R., Hammond M.S.L., Coghill L.S., Wen H., Rusinova R., Clark A.G., Levitan I.B., Shipston M.J. (2001). Alternative splicing switches potassium channel sensitivity to protein phosphorylation. J. Biol. Chem..

[55] Zhou X.B., Arntz C., Kamm S., Motejlek K., Sausbier U., Wang G.X., Ruth P., Korth M. (2001). A molecular switch for specific stimulation of the BKCa channel by cGMP and cAMP kinase. J. Biol. Chem..

[56] Zhou X.-B., Wulfsen I., Utku E., Sausbier U., Sausbier M., Wieland T., Ruth P., Korth M. (2010). Dual role of protein kinase C on BK channel regulation. Proc. Natl. Acad. Sci. USA.

[57] Weiger T.M., Hermann A., Levitan I.B. (2002). Modulation of calcium-activated potassium channels. J. Comp. Physiol. Neuroethol. Sens. Neural. Behav. Physiol..

[58] Alioua A., Kumar Y., Eghbali M., Stefani E. (2006). MaxiK channel partners: Physiological impact. J. Physiol..

[59] Hou S., Heinemann S.H., Hoshi T. (2009). Modulation of BKCa channel gating by endogenous signaling molecules. Physiology.

[60] Sitdikova G.F., Fuchs R., Kainz V., Weiger T.M., Hermann A. (2014). Phosphorylation of BK channels modulates the sensitivity to hydrogen sulfide (H2S). Front. Physiol..

[61] Tang X.D., Garcia M.L., Heinemann S.H., Hoshi T. (2004). Reactive oxygen species impair Slo1 BK channel function by altering cysteine-mediated calcium sensing. Nat. Struct. Mol. Biol..

[62] Tang G., Wu L., Wang R. (2010). Interaction of hydrogen sulfide with ion channels. Clin. Exp. Pharmacol. Physiol..

[63] Wilkinson W.J., Kemp P.J. (2011). Carbon monoxide: An emerging regulator of ion channels. J. Physiol..

[64] Peers C., Bauer C.C., Boyle J.P., Scragg J.L., Dallas M.L. (2012). Modulation of ion channels by hydrogen sulfide. Antioxid. Redox Signal..

[65] Weiger T.M., Holmqvist M.H., Levitan I.B., Clark F.T., Sprague S., Huang W.J., Ge P., Wang C., Lawson D., Jurman M.E. (2000). A novel nervous system beta subunit that downregulates human large conductance calcium-dependent potassium channels. J. Neurosci..

[66] Zhang J., Yan J. (2014). Regulation of BK channels by auxiliary γ subunits. Membr. Physiol. Membr. Biophys..

[67] Torres Y.P., Granados S.T., Latorre R. (2014). Pharmacological consequences of the coexpression of BK channel α and auxiliary β subunits. Membr. Physiol. Membr. Biophys..

[68] McCormack T., McCormack K. (1994). Shaker K^+^ channel beta subunits belong to an NAD(P)H-dependent oxidoreductase superfamily. Cell.

[69] Gulbis J.M., Mann S., MacKinnon R. (1999). Structure of a voltage-dependent K^+^ channel beta subunit. Cell.

[70] Wallner M., Meera P., Toro L. (1996). Determinant for beta-subunit regulation in high-conductance voltage-activated and Ca^2+^-sensitive K^+^ channels: An additional transmembrane region at the N terminus. Proc. Natl. Acad. Sci. USA.

[71] Hanner M., Schmalhofer W.A., Munujos P., Knaus H.G., Kaczorowski G.J., Garcia M.L. (1997). The beta subunit of the high-conductance calcium-activated potassium channel contributes to the high-affinity receptor for charybdotoxin. Proc. Natl. Acad. Sci. USA.

[72] Meera P., Wallner M., Jiang Z., Toro L. (1996). A calcium switch for the functional coupling between alpha (hslo) and beta subunits (KV,Ca beta) of maxi K channels. FEBS Lett..

[73] Orio P., Rojas P., Ferreira G., Latorre R. (2002). New disguises for an old channel: MaxiK channel beta-subunits. News Physiol. Sci..

[74] Wallner M., Meera P., Toro L. (1999). Molecular basis of fast inactivation in voltage and Ca^2+^-activated K^+^ channels: A transmembrane beta-subunit homolog. Proc. Natl. Acad. Sci. USA.

[75] Tseng-Crank J., Godinot N., Johansen T.E., Ahring P.K., Strøbaek D., Mertz R., Foster C.D., Olesen S.P., Reinhart P.H. (1996). Cloning, expression, and distribution of a Ca^2+^-activated K^+^ channel beta-subunit from human brain. Proc. Natl. Acad. Sci. USA.

[76] Bentrop D., Beyermann M., Wissmann R., Fakler B. (2001). NMR structure of the “ball-and-chain” domain of KCNMB2, the beta 2-subunit of large conductance Ca^2+^- and voltage-activated potassium channels. J. Biol. Chem..

[77] Xia X.-M., Ding J.P., Lingle C.J. (2003). Inactivation of BK channels by the NH2 terminus of the beta2 auxiliary subunit: An essential role of a terminal peptide segment of three hydrophobic residues. J. Gen. Physiol..

[78] Ha T.S., Heo M.-S., Park C.-S. (2004). Functional effects of auxiliary beta4-subunit on rat large-conductance Ca^2+^-activated K^+^ channel. Biophys. J..

[79] Brenner R., Chen Q.H., Vilaythong A., Toney G.M., Noebels J.L., Aldrich R.W. (2005). BK channel beta4 subunit reduces dentate gyrus excitability and protects against temporal lobe seizures. Nat. Neurosci..

[80] Brenner R., Jegla T.J., Wickenden A., Liu Y., Aldrich R.W. (2000). Cloning and functional characterization of novel large conductance calcium-activated potassium channel β subunits, hKCNMB3 and hKCNMB4. J. Biol. Chem..

[81] Meera P., Wallner M., Toro L. (2000). A neuronal beta subunit (KCNMB4) makes the large conductance, voltage- and Ca^2+^-activated K^+^ channel resistant to charybdotoxin and iberiotoxin. Proc. Natl. Acad. Sci. USA.

[82] Behrens R., Nolting A., Reimann F., Schwarz M., Waldschütz R., Pongs O. (2000). hKCNMB3 and hKCNMB4, cloning and characterization of two members of the large-conductance calcium-activated potassium channel beta subunit family. FEBS Lett..

[83] Yan J., Aldrich R.W. (2010). LRRC26 auxiliary protein allows BK channel activation at resting voltage without calcium. Nature.

[84] Yan J., Aldrich R.W. (2012). BK potassium channel modulation by leucine-rich repeat-containing proteins. Proc. Natl. Acad. Sci. USA.

[85] Schopperle W.M., Holmqvist M.H., Zhou Y., Wang J., Wang Z., Griffith L.C., Keselman I., Kusinitz F., Dagan D., Levitan I.B. (1998). Slob, a novel protein that interacts with the Slowpoke calcium-dependent potassium channel. Neuron.

[86] Zhou Y., Schopperle W.M., Murrey H., Jaramillo A., Dagan D., Griffith L.C., Levitan I.B. (1999). A dynamically regulated 14–3–3, Slob, and Slowpoke potassium channel complex in *Drosophila* presynaptic nerve terminals. Neuron.

[87] Zeng H., Weiger T.M., Fei H., Levitan I.B. (2006). Mechanisms of two modulatory actions of the channel-binding protein Slob on the *Drosophila* Slowpoke calcium-dependent potassium channel. J. Gen. Physiol..

[88] Zeng H., Weiger T.M., Fei H., Jaramillo A.M., Levitan I.B. (2005). The amino terminus of Slob, Slowpoke channel binding protein, critically influences its modulation of the channel. J. Gen. Physiol..

[89] Jaramillo A.M., Zheng X., Zhou Y., Amado D.A., Sheldon A., Sehgal A., Levitan I.B. (2004). Pattern of distribution and cycling of SLOB, Slowpoke channel binding protein, in *Drosophila*. BMC Neurosci..

[90] Kawakubo T., Naruse K., Matsubara T., Hotta N., Sokabe M. (1999). Characterization of a newly found stretch-activated KCa, ATP channel in cultured chick ventricular myocytes. Am. J. Physiol..

[91] Gasull X., Ferrer E., Llobet A., Castellano A., Nicolás J.M., Palés J., Gual A. (2003). Cell membrane stretch modulates the high-conductance Ca^2+^-activated K^+^ channel in bovine trabecular meshwork cells. Invest. Ophthalmol. Vis. Sci..

[92] Tang Q.Y., Qi Z., Naruse K., Sokabe M. (2003). Characterization of a functionally expressed stretch-activated BKca channel cloned from chick ventricular myocytes. J. Membr. Biol..

[93] Wang W., Huang H., Hou D., Liu P., Wei H., Fu X., Niu W. (2010). Mechanosensitivity of STREX-lacking BKCa channels in the colonic smooth muscle of the mouse. Am. J. Physiol. Gastrointest. Liver Physiol..

[94] Pattillo J.M., Yazejian B., DiGregorio D.A., Vergara J.L., Grinnell A.D., Meriney S.D. (2001). Contribution of presynaptic calcium-activated potassium currents to transmitter release regulation in cultured Xenopus nerve-muscle synapses. Neuroscience.

[95] Meredith A.L., Wiler S.W., Miller B.H., Takahashi J.S., Fodor A.A., Ruby N.F., Aldrich R.W. (2006). BK calcium-activated potassium channels regulate circadian behavioral rhythms and pacemaker output. Nat. Neurosci..

[96] Chen L., Jeffries O., Rowe I.C.M., Liang Z., Knaus H.-G., Ruth P., Shipston M.J. (2010). Membrane trafficking of large conductance calcium-activated potassium channels is regulated by alternative splicing of a transplantable, acidic trafficking motif in the RCK1-RCK2 linker. J. Biol. Chem..

[97] Singh H., Stefani E., Toro L. (2012). Intracellular BK(Ca) (iBK(Ca)) channels. J. Physiol..

[98] Li B., Jie W., Huang L., Wei P., Li S., Luo Z., Friedman A.K., Meredith A.L., Han M.-H., Zhu X.-H. (2014). Nuclear BK channels regulate gene expression via the control of nuclear calcium signaling. Nat. Neurosci..

[99] Nardi A., Olesen S.-P. (2008). BK channel modulators: A comprehensive overview. Curr. Med. Chem..

[100] Rolim A.L.R., Lindsey S.C., Kunii I.S., Fujikawa A.M., Soares F.A., Chiamolera M.I., Maciel R.M.B., da Silva M.R.D. (2010). Ion channelopathies in endocrinology: Recent genetic findings and pathophysiological insights. Arq. Bras. Endocrinol. Metabol..

[101] Catterall W.A. (2010). Ion channel voltage sensors: Structure, function, and pathophysiology. Neuron.

[102] Kullmann D.M., Waxman S.G. (2010). Neurological channelopathies: New insights into disease mechanisms and ion channel function: Neurological channelopathies. J. Physiol..

[103] Zhang L., Li X., Zhou R., Xing G. (2006). Possible role of potassium channel, big K in etiology of schizophrenia. Med. Hypotheses.

[104] Laumonnier F., Roger S., Guérin P., Molinari F., M’rad R., Cahard D., Belhadj A., Halayem M., Persico A.M., Elia M. (2006). Association of a functional deficit of the BKCa channel, a synaptic regulator of neuronal excitability, with autism and mental retardation. Am. J. Psychiatry.

[105] Lorenz S., Heils A., Kasper J.M., Sander T. (2007). Allelic association of a truncation mutation of the KCNMB3 gene with idiopathic generalized epilepsy. Am. J. Med. Genet. Part B Neuropsychiatr. Genet..

[106] Du W., Bautista J.F., Yang H., Diez-Sampedro A., You S.-A., Wang L., Kotagal P., Lüders H.O., Shi J., Cui J. (2005). Calcium-sensitive potassium channelopathy in human epilepsy and paroxysmal movement disorder. Nat. Genet..

[107] Kourie J.I. (1998). Interaction of reactive oxygen species with ion transport mechanisms. Am. J. Physiol..

[108] Hoshi T., Heinemann S.H. (2001). Regulation of cell function by methionine oxidation and reduction. J. Physiol..

[109] Tang X.D., Daggett H., Hanner M., Garcia M.L., McManus O.B., Brot N., Weissbach H., Heinemann S.H., Hoshi T. (2001). Oxidative regulation of large conductance calcium-activated potassium channels. J. Gen. Physiol..

[110] Annunziato L., Pannaccione A., Cataldi M., Secondo A., Castaldo P., di Renzo G., Taglialatela M. (2002). Modulation of ion channels by reactive oxygen and nitrogen species: A pathophysiological role in brain aging?. Neurobiol. Aging.

[111] Chinopoulos C., Adam-Vizi V. (2006). Calcium, mitochondria and oxidative stress in neuronal pathology. FEBS J..

[112] Sesti F., Liu S., Cai S.-Q. (2010). Oxidation of potassium channels by ROS: A general mechanism of aging and neurodegeneration?. Trends Cell Biol..

[113] Sahoo N., Hoshi T., Heinemann S.H. (2014). Oxidative modulation of voltage-gated potassium channels. Antioxid. Redox Signal..

[114] Ray P. D., Huang B.-W., Tsuji Y. (2012). Reactive oxygen species (ROS) homeostasis and redox regulation in cellular signaling. Cell. Signal..

[115] Circu M.L., Aw T.Y. (2010). Reactive oxygen species, cellular redox systems, and apoptosis. Free Radic. Biol. Med..

[116] Bertram C., Hass R. (2008). Cellular responses to reactive oxygen species-induced DNA damage and aging. Biol. Chem..

[117] Peers C., Boyle J.P. (2015). Oxidative modulation of K^+^ channels in the central nervous system in neurodegenerative diseases and aging. Antioxid. Redox Signal..

[118] Arnér E.S.J., Holmgren A. (2000). Physiological functions of thioredoxin and thioredoxin reductase. Eur. J. Biochem..

[119] Levine R.L., Mosoni L., Berlett B.S., Stadtman E.R. (1996). Methionine residues as endogenous antioxidants in proteins. Proc. Natl. Acad. Sci. USA.

[120] Barlow R.S., White R.E. (1998). Hydrogen peroxide relaxes porcine coronary arteries by stimulating BKCa channel activity. Am. J. Physiol..

[121] Gutterman D.D., Miura H., Liu Y. (2005). Redox modulation of vascular tone: Focus of potassium channel mechanisms of dilation. Arterioscler. Thromb. Vasc. Biol..

[122] Stowe D.F., Aldakkak M., Camara A.K.S., Riess M.L., Heinen A., Varadarajan S.G., Jiang M.-T. (2006). Cardiac mitochondrial preconditioning by Big Ca^2+^-sensitive K^+^ channel opening requires superoxide radical generation. Am. J. Physiol. Heart Circ. Physiol..

[123] Thuringer D., Findlay I. (1997). Contrasting effects of intracellular redox couples on the regulation of maxi-K channels in isolated myocytes from rabbit pulmonary artery. J. Physiol..

[124] Lang R.J., Harvey J.R., McPhee G.J., Klemm M.F. (2000). Nitric oxide and thiol reagent modulation of Ca^2+^-activated K^+^ (BKCa) channels in myocytes of the guinea-pig taenia caeci. J. Physiol..

[125] Wang Z.-W., Nara M., Wang Y.-X., Kotlikoff M.I. (1997). Redox regulation of large conductance Ca^2+^-activated K^+^ channels in smooth muscle cells. J. Gen. Physiol..

[126] Riesco-Fagundo A.M., Pérez-García M.T., González C., López-López J.R. (2001). O_2_ modulates large-conductance Ca^2+^-dependent K^+^ channels of rat chemoreceptor cells by a membrane-restricted and CO-sensitive mechanism. Circ. Res..

[127] Gong L.-W., Gao T.M., Huang H., Tong Z. (2000). Redox modulation of large conductance calcium-activated potassium channels in CA1 pyramidal neurons from adult rat hippocampus. Neurosci. Lett..

[128] Zhang G., Horrigan F.T. (2005). Cysteine modification alters voltage- and Ca^2+^-dependent gating of large conductance (BK) potassium channels. J. Gen. Physiol..

[129] Sitdikova G.F., Weiger T.M., Hermann A. (2010). Hydrogen sulfide increases calcium-activated potassium (BK) channel activity of rat pituitary tumor cells. Pflüg. Arch..

[130] Haugland R.P., Spence M.T.Z., Johnson I.D. (1996). Handbook of Fluorescent Probes and Research Chemicals.

[131] Brzezinska A.K., Gebremedhin D., Chilian W.M., Kalyanaraman B., Elliott S.J. (2000). Peroxynitrite reversibly inhibits Ca^2+^-activated K^+^ channels in rat cerebral artery smooth muscle cells. Am. J. Physiol. Heart Circ. Physiol..

[132] Brakemeier S., Eichler I., Knorr A., Fassheber T., Köhler R., Hoyer J. (2003). Modulation of Ca^2+^-activated K^+^ channel in renal artery endothelium *in situ* by nitric oxide and reactive oxygen species. Kidney Int..

[133] Jiang C., Haddad G.G. (1994). Oxygen deprivation inhibits a K^+^ channel independently of cytosolic factors in rat central neurons. J. Physiol..

[134] Lewis A., Peers C., Ashford M.L.J., Kemp P.J. (2002). Hypoxia inhibits human recombinant large conductance, Ca^2+^-activated K^+^ (maxi-K) channels by a mechanism which is membrane delimited and Ca^2+^ sensitive. J. Physiol..

[135] DiChiara T.J., Reinhart P.H. (1997). Redox Modulation of hslo Ca^2+^-Activated K^+^ Channels. J. Neurosci..

[136] Soh H., Jung W., Uhm D.Y., Chung S. (2001). Modulation of large conductance calcium-activated potassium channels from rat hippocampal neurons by glutathione. Neurosci. Lett..

[137] Bednarczyk P., Wieckowski M.R., Broszkiewicz M., Skowronek K., Siemen D., Szewczyk A. (2013). Putative structural and functional coupling of the mitochondrial BKCa channel to the respiratory chain. PLoS One.

[138] Moncada S., Palmer R.M., Higgs E.A. (1991). Nitric oxide: Physiology, pathophysiology, and pharmacology. Pharmacol. Rev..

[139] Yoo D., Jupiter R.C., Pankey E.A., Reddy V.G., Edward J.A., Swan K.W., Peak T.C., Mostany R., Kadowitz P.J. (2015). Analysis of cardiovascular responses to the H2S donors Na2S and NaHS in the rat. Am. J. Physiol..

[140] Wang R., Wu L. (1997). The chemical modification of KCa channels by carbon monoxide in vascular smooth muscle cells. J. Biol. Chem..

[141] Basuroy S., Leffler C.W., Parfenova H. (2013). CORM-A1 prevents blood-brain barrier dysfunction caused by ionotropic glutamate receptor-mediated endothelial oxidative stress and apoptosis. Am. J. Physiol. Cell Physiol..

[142] Ryan M.J., Jernigan N.L., Drummond H.A., McLemore G.R., Rimoldi J.M., Poreddy S.R., Gadepalli R.S.V., Stec D.E. (2006). Renal vascular responses to CORM-A1 in the mouse. Pharmacol. Res..

[143] Soni H., Pandya G., Patel P., Acharya A., Jain M., Mehta A.A. (2011). Beneficial effects of carbon monoxide-releasing molecule-2 (CORM-2) on acute doxorubicin cardiotoxicity in mice: Role of oxidative stress and apoptosis. Toxicol. Appl. Pharmacol..

[144] Smith H., Mann B.E., Motterlini R., Poole R.K. (2011). The carbon monoxide-releasing molecule, CORM-3 (RU(CO)_3_ CL(glycinate)), targets respiration and oxidases in *Campylobacter jejuni*, generating hydrogen peroxide. IUBMB Life.

[145] Stadtman E.R., Berlett B.S. (1998). Reactive oxygen-mediated protein oxidation in aging and disease. Drug Metab. Rev..

[146] Manning G. Genomic Overview of Protein Kinases. http://www.wormbook.org.

[147] Levitan I.B. (1985). Phosphorylation of ion channels. J. Membr. Biol..

[148] Park K.-S., Yang J.-W., Seikel E., Trimmer J.S. (2008). Potassium channel phosphorylation in excitable cells: Providing dynamic functional variability to a diverse family of ion channels. Physiology.

[149] Ismailov I.I., Benos D.J. (1995). Effects of phosphorylation on ion channel function. Kidney Int..

[150] Hille B. (1994). Modulation of ion-channel function by G-protein-coupled receptors. Trends Neurosci..

[151] Shipston M.J. (2014). Ion channel regulation by protein S-acylation. J. Gen. Physiol..

[152] Schubert R., Nelson M.T. (2001). Protein kinases: Tuners of the BKCa channel in smooth muscle. Trends Pharmacol. Sci..

[153] Newton P.M., Messing R.O. (2006). Intracellular signaling pathways that regulate behavioral responses to ethanol. Pharmacol. Ther..

[154] Lorca R.A., Prabagaran M., England S.K. (2014). Functional insights into modulation of BKCa channel activity to alter myometrial contractility. Front. Physiol..

[155] Ashcroft F.M., Rorsman P. (2013). K(ATP) channels and islet hormone secretion: New insights and controversies. Nat. Rev. Endocrinol..

[156] Friedman J., Gadoth N., Göbel H.H. (2011). Why Is the Nervous System Vulnerable to Oxidative Stress?. Oxidative Stress and Free Radical Damage in Neurology, Oxidative Stress in Applied Basic Research and Clinical Practice.

[157] Gao T.-M., Fung M.-L. (2002). Decreased large conductance Ca^2+^-activated K^+^ channel activity in dissociated CA1 hippocampal neurons in rats exposed to perinatal and postnatal hypoxia. Neurosci. Lett..

[158] Ji L.L., Fu R., Mitchell E.W. (1992). Glutathione and antioxidant enzymes in skeletal muscle: Effects of fiber type and exercise intensity. J. Appl. Physiol..

[159] Meister A. (1995). Glutathione metabolism. Methods Enzymol..

[160] Langeveld C.H., Schepens E., Jongenelen C.A., Stoof J.C., Hjelle O.P., Ottersen O.P., Drukarch B. (1996). Presence of glutathione immunoreactivity in cultured neurones and astrocytes. Neuroreport.

[161] Dringen R., Kussmaul L., Gutterer J.M., Hirrlinger J., Hamprecht B. (1999). The glutathione system of peroxide detoxification is less efficient in neurons than in astroglial cells. J. Neurochem..

[162] Fernandez-Fernandez S., Almeida A., Bolaños J.P. (2012). Antioxidant and bioenergetic coupling between neurons and astrocytes. Biochem. J..

[163] Martin H.L., Teismann P. (2009). Glutathione—A review on its role and significance in Parkinson’s disease. Fed. Am. Soc. Exp. Biol..

[164] Smeyne M., Smeyne R.J. (2013). Glutathione metabolism and Parkinson’s disease. Free Radic. Biol. Med..

[165] Liu H., Moczydlowski E., Haddad G.G. (1999). O_2_ deprivation inhibits Ca^2+^-activated K^+^ channels via cytosolic factors in mice neocortical neurons. J. Clin. Invest..

[166] Wyatt C.N., Wright C., Bee D., Peers C. (1995). O_2_-sensitive K^+^ currents in carotid body chemoreceptor cells from normoxic and chronically hypoxic rats and their roles in hypoxic chemotransduction. Proc. Natl. Acad. Sci. USA.

[167] Clark A.G., Hall S.K., Shipston M.J. (1999). ATP inhibition of a mouse brain large-conductance K^+^ (mslo) channel variant by a mechanism independent of protein phosphorylation. J. Physiol..

[168] López-López J.R., González C. (1992). Time course of K^+^ current inhibition by low oxygen in chemoreceptor cells of adult rabbit carotid body. Effects of carbon monoxide. FEBS Lett..

[169] Gleichmann M., Mattson M.P. (2011). Neuronal calcium homeostasis and dysregulation. Antioxid. Redox Signal..

[170] Storm J.F. (1987). Action potential repolarization and a fast after-hyperpolarization in rat hippocampal pyramidal cells. J. Physiol..

[171] Hu H., Shao L.R., Chavoshy S., Gu N., Trieb M., Behrens R., Laake P., Pongs O., Knaus H.G., Ottersen O.P. (2001). Presynaptic Ca^2+^-activated K^+^ channels in glutamatergic hippocampal terminals and their role in spike repolarization and regulation of transmitter release. J. Neurosci..

[172] Mancini M., Soldovieri M.V., Gessner G., Wissuwa B., Barrese V., Boscia F., Secondo A., Miceli F., Franco C., Ambrosino P. (2014). Critical role of large-conductance calcium- and voltage-activated potassium channels in leptin-induced neuroprotection of N-methyl-D-aspartate-exposed cortical neurons. Pharmacol. Res..

[173] Rundén-Pran E., Haug F.M., Storm J.F., Ottersen O.P. (2002). BK channel activity determines the extent of cell degeneration after oxygen and glucose deprivation: A study in organotypical hippocampal slice cultures. Neuroscience.

[174] Liao Y., Kristiansen A.-M., Oksvold C.P., Tuvnes F.A., Gu N., Rundén-Pran E., Ruth P., Sausbier M., Storm J.F. (2010). Neuronal Ca^2+^-activated K^+^ channels limit brain infarction and promote survival. PLoS ONE.

[175] Kulawiak B., Szewczyk A. (2012). Glutamate-induced cell death in HT22 mouse hippocampal cells is attenuated by paxilline, a BK channel inhibitor. Mitochondrion.

[176] Ye H., Jalini S., Mylvaganam S., Carlen P. (2010). Activation of large-conductance Ca^2+^-activated K^+^ channels depresses basal synaptic transmission in the hippocampal CA1 area in APP (swe/ind) TgCRND8 mice. Neurobiol. Aging.

[177] Gáspár T., Domoki F., Lenti L., Katakam P.V.G., Snipes J.A., Bari F., Busija D.W. (2009). Immediate neuronal preconditioning by NS1619. Brain Res..

[178] Gáspár T., Katakam P., Snipes J.A., Kis B., Domoki F., Bari F., Busija D.W. (2008). Delayed neuronal preconditioning by NS1619 is independent of calcium activated potassium channels. J. Neurochem..

[179] Xie H., Zhang Y.-Q., Pan X.-L., Wu S.-H., Chen X., Wang J., Liu H., Qian X.-Z., Liu Z.-G., Liu L.-J. (2014). Decreased calcium-activated potassium channels by hypoxia causes abnormal firing in the spontaneous firing medial vestibular nuclei neurons. Eur. Arch. Otorhinolaryngol..

[180] Tang X.D., Xu R., Reynolds M.F., Garcia M.L., Heinemann S.H., Hoshi T. (2003). Haem can bind to and inhibit mammalian calcium-dependent Slo1 BK channels. Nature.

[181] Kulawiak B., Kudin A.P., Szewczyk A., Kunz W.S. (2008). BK channel openers inhibit ROS production of isolated rat brain mitochondria. Exp. Neurol..

[182] Augustynek B., Kudin A.P., Bednarczyk P., Szewczyk A., Kunz W.S. (2014). Hemin inhibits the large conductance potassium channel in brain mitochondria: A putative novel mechanism of neurodegeneration. Exp. Neurol..

[183] Hayabuchi Y., Nakaya Y., Matsuoka S., Kuroda Y. (1998). Hydrogen peroxide-induced vascular relaxation in porcine coronary arteries is mediated by Ca^2+^-activated K^+^ channels. Heart Vessels.

[184] Zeng X.-H., Xia X.-M., Lingle C.J. (2003). Redox-sensitive extracellular gates formed by auxiliary beta subunits of calcium-activated potassium channels. Nat. Struct. Biol..

[185] Gribkoff V.K., Starrett J.E., Dworetzky S.I., Hewawasam P., Boissard C.G., Cook D.A., Frantz S.W., Heman K., Hibbard J.R., Huston K. (2001). Targeting acute ischemic stroke with a calcium-sensitive opener of maxi-K potassium channels. Nat. Med..

[186] Shintani Y., Node K., Asanuma H., Sanada S., Takashima S., Asano Y., Liao Y., Fujita M., Hirata A., Shinozaki Y. (2004). Opening of Ca^2+^-activated K^+^ channels is involved in ischemic preconditioning in canine hearts. J. Mol. Cell. Cardiol..

[187] Bentzen B.H., Olesen S.-P., Rønn L.C.B., Grunnet M. (2014). BK channel activators and their therapeutic perspectives. Front. Physiol..

[188] Wojtovich A.P., Nadtochiy S.M., Urciuoli W.R., Smith C.O., Grunnet M., Nehrke K., Brookes P.S. (2013). A non-cardiomyocyte autonomous mechanism of cardioprotection involving the SLO1 BK channel. Peer J..

[189] Wagner S., Rokita A.G., Anderson M.E., Maier L.S. (2013). Redox regulation of sodium and calcium handling. Antioxid. Redox Signal..

[190] Jovanović S., Crawford R.M., Ranki H.J., Jovanović A. (2003). Large conductance Ca^2+^-activated K^+^ channels sense acute changes in oxygen tension in alveolar epithelial cells. Am. J. Respir. Cell Mol. Biol..

[191] Williams S.E.J., Wootton P., Mason H.S., Bould J., Iles D.E., Riccardi D., Peers C., Kemp P.J. (2004). Hemoxygenase-2 is an oxygen sensor for a calcium-sensitive potassium channel. Science.

[192] Sheu S.-J., Wu S.-N. (2003). Mechanism of inhibitory actions of oxidizing agents on calcium-activated potassium current in cultured pigment epithelial cells of the human retina. Invest. Ophthalmol. Vis. Sci..

[193] Mathias S., Peña L.A., Kolesnick R.N. (1998). Signal transduction of stress via ceramide. Biochem. J..

[194] Li X., Becker K.A., Zhang Y. (2010). Ceramide in redox signaling and cardiovascular diseases. Cell. Physiol. Biochem..

[195] Li P.L., Zhang D.X., Zou A.P., Campbell W.B. (1999). Effect of ceramide on KCa channel activity and vascular tone in coronary arteries. Hypertension.

[196] Hanner M., Vianna-Jorge R., Kamassah A., Schmalhofer W.A., Knaus H.G., Kaczorowski G.J., Garcia M.L. (1998). The beta subunit of the high conductance calcium-activated potassium channel. Identification of residues involved in charybdotoxin binding. J. Biol. Chem..

[197] Knaus H.G., Folander K., Garcia-Calvo M., Garcia M.L., Kaczorowski G.J., Smith M., Swanson R. (1994). Primary sequence and immunological characterization of beta-subunit of high conductance Ca^2+^-activated K^+^ channel from smooth muscle. J. Biol. Chem..

[198] Santarelli L.C., Chen J., Heinemann S.H., Hoshi T. (2004). The beta1 subunit enhances oxidative regulation of large-conductance calcium-activated K^+^ channels. J. Gen. Physiol..

[199] Santarelli L.C., Wassef R., Heinemann S.H., Hoshi T. (2006). Three methionine residues located within the regulator of conductance for K^+^ (RCK) domains confer oxidative sensitivity to large-conductance Ca^2+^-activated K^+^ channels. J. Physiol..

[200] Lu T., Wang X.-L., He T., Zhou W., Kaduce T.L., Katusic Z.S., Spector A.A., Lee H.-C. (2005). Impaired arachidonic acid-mediated activation of large-conductance Ca^2+^-activated K^+^ channels in coronary arterial smooth muscle cells in Zucker Diabetic Fatty rats. Diabetes.

[201] Lu T., He T., Katusic Z.S., Lee H.-C. (2006). Molecular mechanisms mediating inhibition of human large conductance Ca^2+^-activated K^+^ channels by high glucose. Circ. Res..

[202] Lu T., Zhang D.-M., Wang X.-L., He T., Wang R.-X., Chai Q., Katusic Z.S., Lee H.-C. (2010). Regulation of coronary arterial BK channels by caveolae-mediated angiotensin II signaling in diabetes mellitus. Circ. Res..

[203] Burgoyne J.R., Madhani M., Cuello F., Charles R.L., Brennan J.P., Schröder E., Browning D.D., Eaton P. (2007). Cysteine redox sensor in PKGIa enables oxidant-induced activation. Science.

[204] Zhang D.X., Borbouse L., Gebremedhin D., Mendoza S.A., Zinkevich N.S., Li R., Gutterman D.D. (2012). H2O2-induced dilation in human coronary arterioles: Role of protein kinase G dimerization and large-conductance Ca^2+^-activated K^+^ channel activation. Circ. Res..

[205] Rodgers-Garlick C.I., Hogg D.W., Buck L.T. (2013). Oxygen-sensitive reduction in Ca^2+^-activated K^+^ channel open probability in turtle cerebrocortex. Neuroscience.

[206] O’Reilly J.P., Cummins T.R., Haddad G.G. (1997). Oxygen deprivation inhibits Na^+^ current in rat hippocampal neurones via protein kinase C. J. Physiol..

[207] Gebremedhin D., Bonnet P., Greene A.S., England S.K., Rusch N.J., Lombard J.H., Harder D.R. (1994). Hypoxia increases the activity of Ca^2+^-sensitive K^+^ channels in cat cerebral arterial muscle cell membranes. Pflüg. Arch..

[208] Gebremedhin D., Yamaura K., Harder D.R. (2008). Role of 20-HETE in the hypoxia-induced activation of Ca^2+^-activated K^+^ channel currents in rat cerebral arterial muscle cells. Am. J. Physiol. Heart Circ. Physiol..

[209] Hu L.-F., Lu M., Hon Wong P.T., Bian J.-S. (2011). Hydrogen sulfide: Neurophysiology and neuropathology. Antioxid. Redox Signal..

[210] Hu X.-Q., Xiao D., Zhu R., Huang X., Yang S., Wilson S.M., Zhang L. (2012). Chronic hypoxia suppresses pregnancy-induced upregulation of large-conductance Ca^2+^-activated K^+^ channel activity in uterine arteries. Hypertension.

[211] Zhu R., Xiao D., Zhang L. (2013). Potassium channels and uterine vascular adaptation to pregnancy and chronic hypoxia. Curr. Vasc. Pharmacol..

[212] Xiao D., Zhu R., Zhang L. (2014). Gestational hypoxia up-regulates protein kinase C and inhibits calcium-activated potassium channels in ovine uterine arteries. Int. J. Med. Sci..

[213] Wong C.-M., Tsang S.-Y., Yao X., Chan F.L., Huang Y. (2008). Differential effects of estrogen and progesterone on potassium channels expressed in Xenopus oocytes. Steroids.

[214] Zhu R., Huang X., Hu X.-Q., Xiao D., Zhang L. (2014). Gestational hypoxia increases reactive oxygen species and inhibits steroid hormone-mediated upregulation of Ca^2+^-activated K^+^ channel function in uterine arteries. Hypertension.

[215] Wareing M. (2014). Oxygen sensitivity, potassium channels, and regulation of placental vascular tone. Microcirculation.

[216] Szewczyk A., Skalska J., Głab M., Kulawiak B., Malińska D., Koszela-Piotrowska I., Kunz W.S. (2006). Mitochondrial potassium channels: From pharmacology to function. Biochim. Biophys. Acta.

[217] Szewczyk A., Jarmuszkiewicz W., Kunz W.S. (2009). Mitochondrial potassium channels. IUBMB Life.

[218] Kalogeris T., Bao Y., Korthuis R.J. (2014). Mitochondrial reactive oxygen species: A double edged sword in ischemia/reperfusion *vs*. preconditioning. Redox Biol..

[219] Balderas E., Zhang J., Stefani E., Toro L. (2015). Mitochondrial BKCa channel. Front. Physiol..

[220] Siemen D., Loupatatzis C., Borecky J., Gulbins E., Lang F. (1999). Ca^2+^-activated K channel of the BK-type in the inner mitochondrial membrane of a human glioma cell line. Biochem. Biophys. Res. Commun..

[221] Xu W., Liu Y., Wang S., McDonald T., van Eyk J.E., Sidor A., O’Rourke B. (2002). Cytoprotective role of Ca^2+^- activated K^+^ channels in the cardiac inner mitochondrial membrane. Science.

[222] Singh H., Lu R., Bopassa J.C., Meredith A.L., Stefani E., Toro L. (2013). MitoBK(Ca) is encoded by the Kcnma1 gene, and a splicing sequence defines its mitochondrial location. Proc. Natl. Acad. Sci. USA.

[223] Heinen A., Camara A.K.S., Aldakkak M., Rhodes S.S., Riess M.L., Stowe D.F. (2007). Mitochondrial Ca^2+^-induced K^+^ influx increases respiration and enhances ROS production while maintaining membrane potential. Am. J. Physiol. Cell Physiol..

[224] Heinen A., Aldakkak M., Stowe D.F., Rhodes S.S., Riess M.L., Varadarajan S.G., Camara A.K.S. (2007). Reverse electron flow-induced ROS production is attenuated by activation of mitochondrial Ca^2+^-sensitive K^+^ channels. Am. J. Physiol. Heart Circ. Physiol..

[225] Bentzen B.H., Osadchii O., Jespersen T., Hansen R.S., Olesen S.-P., Grunnet M. (2009). Activation of big conductance Ca^2+^-activated K^+^ channels (BK) protects the heart against ischemia-reperfusion injury. Pflüg. Arch..

[226] Sakamoto K., Ohya S., Muraki K., Imaizumi Y. (2008). A novel opener of large-conductance Ca^2+^-activated K^+^ (BK) channel reduces ischemic injury in rat cardiac myocytes by activating mitochondrial K(Ca) channel. J. Pharmacol. Sci..

[227] Ohya S., Kuwata Y., Sakamoto K., Muraki K., Imaizumi Y. (2005). Cardioprotective effects of estradiol include the activation of large-conductance Ca^2+^-activated K^+^ channels in cardiac mitochondria. Am. J. Physiol. Heart Circ. Physiol..

[228] Tano J.-Y., Gollasch M. (2014). Calcium-activated potassium channels in ischemia reperfusion: A brief update. Front. Physiol..

[229] Hagen B.M., Sanders K.M. (2006). Deglycosylation of the beta1-subunit of the BK channel changes its biophysical properties. Am. J. Physiol. Cell Physiol..

[230] Borchert G.H., Yang C., Kolár F. (2011). Mitochondrial BKCa channels contribute to protection of cardiomyocytes isolated from chronically hypoxic rats. Am. J. Physiol. Heart Circ. Physiol..

[231] Borchert G.H., Hlaváčková M., Kolář F. (2013). Pharmacological activation of mitochondrial BK(Ca) channels protects isolated cardiomyocytes against simulated reperfusion-induced injury. Exp. Biol. Med. Maywood.

[232] Neckár J., Borchert G.H., Hlousková P., Mícová P., Nováková O., Novák F., Hroch M., Papousek F., Ost’ádal B., Kolár F. (2013). Brief daily episode of normoxia inhibits cardioprotection conferred by chronic continuous hypoxia. Role of oxidative stress and BKCa channels. Curr. Pharm. Des..

[233] Noursadeghi E., Jafari A., Saghiri R., Sauve R., Eliassi A. (2014). Impairment of brain mitochondrial charybdotoxin- and ATP-insensitive BK channel activities in diabetes. Neuromol. Med..

[234] Leffler C.W., Parfenova H., Jaggar J.H. (2011). Carbon monoxide as an endogenous vascular modulator. Am. J. Physiol. Heart Circ. Physiol..

[235] Li H., Förstermann U. (2009). Prevention of atherosclerosis by interference with the vascular nitric oxide system. Curr. Pharm. Des..

[236] Ooi L., Gigout S., Pettinger L., Gamper N. (2013). Triple cysteine module within M-type K^+^ channels mediates reciprocal channel modulation by nitric oxide and reactive oxygen species. J. Neurosci..

[237] Liu Y., Gutterman D.D. (2002). Oxidative stress and potassium channel function. Clin. Exp. Pharmacol. Physiol..

[238] Drews G., Düfer M. (2012). Role of K(ATP) channels in β-cell resistance to oxidative stress. Diabetes Obes. MeTable.

[239] Liu Y., Kalogeris T., Wang M., Zuidema M.Y., Wang Q., Dai H., Davis M.J., Hill M.A., Korthuis R.J. (2012). Hydrogen sulfide preconditioning or neutrophil depletion attenuates ischemia-reperfusion-induced mitochondrial dysfunction in rat small intestine. Am. J. Physiol. Gastrointest. Liver Physiol..

[240] Moudgil R., Michelakis E.D., Archer S.L. (2006). The role of K^+^ channels in determining pulmonary vascular tone, oxygen sensing, cell proliferation, and apoptosis: Implications in hypoxic pulmonary vasoconstriction and pulmonary arterial hypertension. Microcirculation.

[241] Wolfram Kuhlmann C.R., Wiebke Lüdders D., Schaefer C.A., Kerstin Most A., Backenköhler U., Neumann T., Tillmanns H., Erdogan A. (2004). Lysophosphatidylcholine-induced modulation of Ca^2+^-activated K^+^ channels contributes to ROS-dependent proliferation of cultured human endothelial cells. J. Mol. Cell. Cardiol..

[242] Pardo L.A. (2004). Voltage-gated potassium channels in cell proliferation. Physiology.

[243] Huang X., Jan L.Y. (2014). Targeting potassium channels in cancer. J. Cell Biol..

[244] Pardo L.A., Stühmer W. (2014). The roles of K^+^ channels in cancer. Nat. Rev. Cancer.

[245] Yang M., Brackenbury W.J. (2013). Membrane potential and cancer progression. Front. Physiol..

[246] Barghouth P.G., Thiruvalluvan M., Oviedo N.J. (2015). Bioelectrical regulation of cell cycle and the planarian model system. Biochim. Biophys. Acta.

[247] Blackiston D.J., McLaughlin K.A., Levin M. (2009). Bioelectric controls of cell proliferation: Ion channels, membrane voltage and the cell cycle. Cell Cycle.

[248] Weiger T.M., Hermann A. (2014). Cell proliferation, potassium channels, polyamines and their interactions: A mini review. Amino Acids.

[249] Chattopadhyay M.K., Tabor C.W., Tabor H. (2006). Polyamine deficiency leads to accumulation of reactive oxygen species in a spe2Delta mutant of Saccharomyces cerevisiae. Yeast Chichester. Engl..

[250] Takano K., Ogura M., Nakamura Y., Yoneda Y. (2005). Neuronal and glial responses to polyamines in the ischemic brain. Curr. Neurovasc. Res..

[251] Pamenter M.E., Haddad G.G. (2014). Do BK channels mediate glioma hypoxia-tolerance?. Channels.

[252] Liu X., Chang Y., Reinhart P.H., Sontheimer H., Chang Y. (2002). Cloning and characterization of glioma BK, a novel BK channel isoform highly expressed in human glioma cells. J. Neurosci..

[253] Sontheimer H. (2008). An unexpected role for ion channels in brain tumor metastasis. Exp. Biol. Med..

[254] Cheng Y., Gu X.Q., Bednarczyk P., Wiedemann F.R., Haddad G.G., Siemen D. (2008). Hypoxia increases activity of the BK-channel in the inner mitochondrial membrane and reduces activity of the permeability transition pore. Cell. Physiol. Biochem..

[255] Gu X.Q., Pamenter M.E., Siemen D., Sun X., Haddad G.G. (2014). Mitochondrial but not plasmalemmal BK channels are hypoxia-sensitive in human glioma. Glia.

[256] Gu X.Q., Siemen D., Parvez S., Cheng Y., Xue J., Zhou D., Sun X., Jonas E.A., Haddad G.G. (2007). Hypoxia increases BK channel activity in the inner mitochondrial membrane. Biochem. Biophys. Res. Commun..

[257] Han X., Xi L., Wang H., Huang X., Ma X., Han Z., Wu P., Ma X., Lu Y., Wang G. (2008). The potassium ion channel opener NS1619 inhibits proliferation and induces apoptosis in A2780 ovarian cancer cells. Biochem. Biophys. Res. Commun..

[258] Debska-Vielhaber G., Godlewski M.M., Kicinska A., Skalska J., Kulawiak B., Piwonska M., Zablocki K., Kunz W.S., Szewczyk A., Motyl T. (2009). Large-conductance K^+^ channel openers induce death of human glioma cells. J. Physiol. Pharmacol..

[259] Halestrap A.P. (2009). What is the mitochondrial permeability transition pore?. J. Mol. Cell. Cardiol..

[260] Chatterjee S., Nieman G.F., Christie J.D., Fisher A.B. (2014). Shear stress-related mechanosignaling with lung ischemia: Lessons from basic research can inform lung transplantation. Am. J. Physiol. Lung Cell. Mol. Physiol..

[261] Mohamed M.S.A. (2015). Calcium-activated potassium channels in ischemia-reperfusion: Learning for the clinical application. Pulm. Med..

[262] Noda K., Shigemura N., Tanaka Y., Bhama J., D’Cunha J., Kobayashi H., Luketich J.D., Bermudez C.A. (2014). Hydrogen preconditioning during *ex vivo* lung perfusion improves the quality of lung grafts in rats. Transplantation.

[263] Dong D.-L., Zhang Y., Lin D.-H., Chen J., Patschan S., Goligorsky M.S., Nasjletti A., Yang B.-F., Wang W.-H. (2007). Carbon monoxide stimulates the Ca^2+^-activated big conductance K channels in cultured human endothelial cells. Hypertension.

[264] Carrasquel-Ursulaez W., Contreras G.F., Sepúlveda R.V., Aguayo D., González-Nilo F., González C., Latorre R. (2015). Hydrophobic interaction between contiguous residues in the S6 transmembrane segment acts as a stimuli integration node in the BK channel. J. Gen. Physiol..

[265] McBride C.S., Baier F., Omondi A.B., Spitzer S.A., Lutomiah J., Sang R., Ignell R., Vosshall L.B. (2014). Evolution of mosquito preference for humans linked to an odorant receptor. Nature.

[266] Adler E.M. (2015). Of ELIC and evolution. J. Gen. Physiol..

[267] Korovkina V.P., England S.K. (2002). Molecular diversity of vascular potassium channel isoforms. Clin. Exp. Pharmacol. Physiol..

[268] Ko E.A., Han J., Jung I.D., Park W.S. (2008). Physiological roles of K^+^ channels in vascular smooth muscle cells. J. Smooth Muscle Res..

[269] Park S.W., Noh H.J., Sung D.J., Kim J.G., Kim J.M., Ryu S.-Y., Kang K., Kim B., Bae Y.M., Cho H. (2015). Hydrogen peroxide induces vasorelaxation by enhancing 4-aminopyridine-sensitive Kv currents through S-glutathionylation. Pflüg. Arch..

[270] Fukumoto M., Nakaizumi A., Zhang T., Lentz S.I., Shibata M., Puro D.G. (2012). Vulnerability of the retinal microvasculature to oxidative stress: Ion channel-dependent mechanisms. Am. J. Physiol. Cell Physiol..

